# Macrophage-specific circular RNA circHIPK2, inflammation, and fibrosis after myocardial infarction

**DOI:** 10.1093/eurheartj/ehaf1116

**Published:** 2026-02-11

**Authors:** Mira Jung, Arne Schmidt, Marida Sansonetti, Bashar Al soodi, Sabrina Thum, Angelika Pfanne, Annette Just, Ke Xiao, Cheng-Kai Huang, Sergej Erschow, Melanie Ricke-Hoch, Lea Oehlsen, Wilson Agyapong, Karina Jansen, Jonas Blume, Gwen Büchler, Erika Hilbold, Christian Bär, Natalie Weber, Maximilian Schinke, Ariane Hai Ha Nguyen, Kai C Wollert, James T Thackeray, Frank Bengel, Nico Lachmann, Thomas Thum

**Affiliations:** Institute of Molecular and Translational Therapeutic Strategies, Hannover Medical School, Carl-Neuberg-Str. 1, Hannover 30625, Germany; Institute of Molecular and Translational Therapeutic Strategies, Hannover Medical School, Carl-Neuberg-Str. 1, Hannover 30625, Germany; Institute of Molecular and Translational Therapeutic Strategies, Hannover Medical School, Carl-Neuberg-Str. 1, Hannover 30625, Germany; Institute of Molecular and Translational Therapeutic Strategies, Hannover Medical School, Carl-Neuberg-Str. 1, Hannover 30625, Germany; Institute of Molecular and Translational Therapeutic Strategies, Hannover Medical School, Carl-Neuberg-Str. 1, Hannover 30625, Germany; Institute of Molecular and Translational Therapeutic Strategies, Hannover Medical School, Carl-Neuberg-Str. 1, Hannover 30625, Germany; Institute of Molecular and Translational Therapeutic Strategies, Hannover Medical School, Carl-Neuberg-Str. 1, Hannover 30625, Germany; Institute of Molecular and Translational Therapeutic Strategies, Hannover Medical School, Carl-Neuberg-Str. 1, Hannover 30625, Germany; Preclinical Pharmacology and Toxicology, Fraunhofer Institute for Toxicology and Experimental Medicine (ITEM), Nikolai-Fuchs-Straße 1, Hannover 30625, Germany; Institute of Molecular and Translational Therapeutic Strategies, Hannover Medical School, Carl-Neuberg-Str. 1, Hannover 30625, Germany; Department of Cardiology and Angiology, Hannover Medical School, Carl-Neuberg-Str. 1, Hannover 30625, Germany; Department of Cardiology and Angiology, Hannover Medical School, Carl-Neuberg-Str. 1, Hannover 30625, Germany; Institute of Molecular and Translational Therapeutic Strategies, Hannover Medical School, Carl-Neuberg-Str. 1, Hannover 30625, Germany; Preclinical Pharmacology and Toxicology, Fraunhofer Institute for Toxicology and Experimental Medicine (ITEM), Nikolai-Fuchs-Straße 1, Hannover 30625, Germany; Institute of Molecular and Translational Therapeutic Strategies, Hannover Medical School, Carl-Neuberg-Str. 1, Hannover 30625, Germany; Institute of Molecular and Translational Therapeutic Strategies, Hannover Medical School, Carl-Neuberg-Str. 1, Hannover 30625, Germany; Institute of Molecular and Translational Therapeutic Strategies, Hannover Medical School, Carl-Neuberg-Str. 1, Hannover 30625, Germany; Institute of Molecular and Translational Therapeutic Strategies, Hannover Medical School, Carl-Neuberg-Str. 1, Hannover 30625, Germany; Institute of Molecular and Translational Therapeutic Strategies, Hannover Medical School, Carl-Neuberg-Str. 1, Hannover 30625, Germany; Institute of Molecular and Translational Therapeutic Strategies, Hannover Medical School, Carl-Neuberg-Str. 1, Hannover 30625, Germany; Preclinical Pharmacology and Toxicology, Fraunhofer Institute for Toxicology and Experimental Medicine (ITEM), Nikolai-Fuchs-Straße 1, Hannover 30625, Germany; Fraunhofer Cluster of Excellence Immune-Mediated Diseases (CIMD), Nikolai-Fuchs-Straße 1, Hannover 30625, Germany; Center for Translational Regenerative Medicine, Hannover Medical School, Carl-Neuberg-Str. 1, Hannover 30625, Germany; Institute of Molecular and Translational Therapeutic Strategies, Hannover Medical School, Carl-Neuberg-Str. 1, Hannover 30625, Germany; Dean’s Office for Academic Career Development, nextGENERATION Medical Scientist Program, Hannover Medical School, Carl-Neuberg-Str. 1, Hannover 30625, Germany; Preclinical Pharmacology and Toxicology, Fraunhofer Institute for Toxicology and Experimental Medicine (ITEM), Nikolai-Fuchs-Straße 1, Hannover 30625, Germany; Department of Pediatric Pneumology, Allergology and Neonatology, Hannover Medical School, Carl-Neuberg-Str. 1, Hannover 30625, Germany; Department of Cardiology and Angiology, Hannover Medical School, Carl-Neuberg-Str. 1, Hannover 30625, Germany; Division of Molecular and Translational Cardiology, Hannover Medical School, Carl-Neuberg-Str. 1, Hannover 30625, Germany; Department of Nuclear Medicine, Hannover Medical School, Carl-Neuberg-Str. 1, Hannover 30625, Germany; Department of Nuclear Medicine, Hannover Medical School, Carl-Neuberg-Str. 1, Hannover 30625, Germany; Preclinical Pharmacology and Toxicology, Fraunhofer Institute for Toxicology and Experimental Medicine (ITEM), Nikolai-Fuchs-Straße 1, Hannover 30625, Germany; Center for Translational Regenerative Medicine, Hannover Medical School, Carl-Neuberg-Str. 1, Hannover 30625, Germany; Department of Pediatric Pneumology, Allergology and Neonatology, Hannover Medical School, Carl-Neuberg-Str. 1, Hannover 30625, Germany; Institute of Molecular and Translational Therapeutic Strategies, Hannover Medical School, Carl-Neuberg-Str. 1, Hannover 30625, Germany; Center for Translational Regenerative Medicine, Hannover Medical School, Carl-Neuberg-Str. 1, Hannover 30625, Germany

**Keywords:** Myocardial infarction, Circular RNA, Macrophages, Inflammation, Immune therapy, RNA therapy

## Abstract

**Background and Aims:**

Cardiac remodelling following MI is intricately linked to macrophage polarization dynamics, yet the underlying mechanisms remain incompletely elucidated. In this study, circHIPK2, a novel circRNA, was identified as being upregulated in inflammatory cardiac macrophages following MI. Its expression correlated with post-MI inflammation dynamics, suggesting a regulatory role in macrophage polarization. It was hypothesized that circHIPK2 functions as a molecular switch of macrophage polarization during MI progression.

**Methods:**

To test this hypothesis, circHIPK2 levels were modulated *in vitro* using siRNA and overexpression vectors. A murine MI model with macrophage-targeted inhibition of circHIPK2 through AAV9-mediated shRNA delivery was employed. Cardiac function was evaluated using echocardiography, histology, and PET imaging. An *ex vivo* co-culture platform incorporating living myocardial slices from heart failure patients and circHIPK2-modulated human iPSC-derived macrophages was established to explore a translational potential.

**Results:**

CircHIPK2 interacts with its binding partner G3BP1 to promote stress granule formation in macrophages. This interaction initiates a downstream inflammatory cascade, highlighting its role in immune regulation. Inhibition of circHIPK2 suppressed inflammatory signalling and reduced pro-inflammatory cytokine secretion. In a mouse model of MI, macrophage-specific inhibition of circHIPK2 improved cardiac function, reshaped the inflammatory environment, and attenuated fibrosis progression. Silencing circHIPK2 in human macrophages demonstrated therapeutic potential in established heart failure, promoting beneficial cardiac healing.

**Conclusions:**

Targeting circHIPK2 in macrophages may represent a promising therapeutic strategy for treating inflammatory cardiac conditions and heart failure, potentially leading to the development of RNA-based therapies targeting immune cells in MI treatment.

## Structured Graphical Abstract

**Structured Graphical Abstract:**
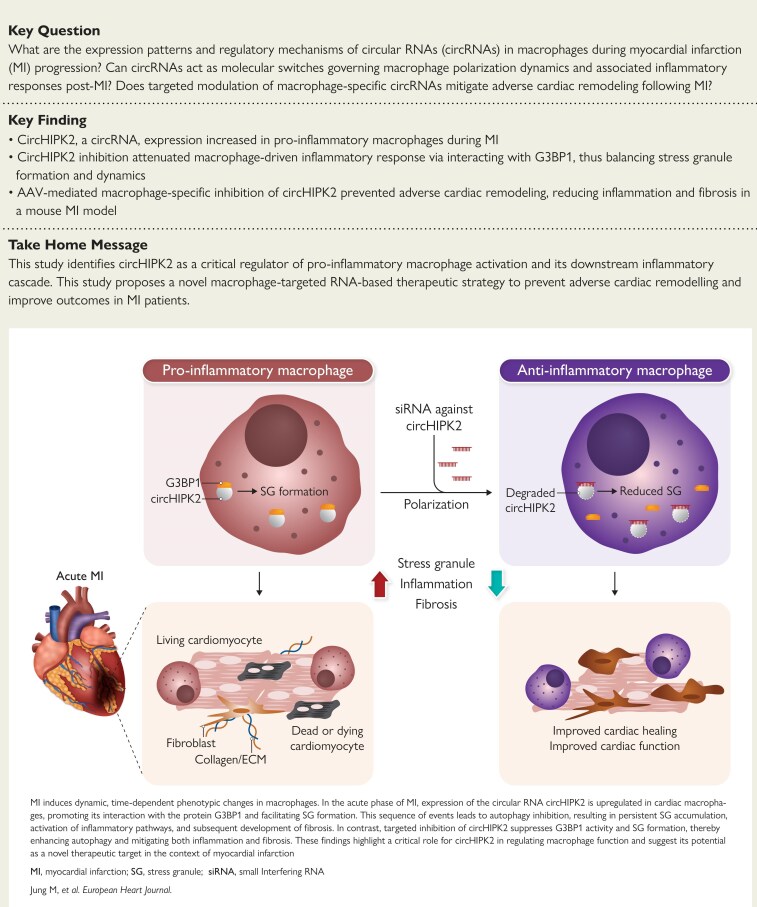

**See the editorial comment for this article ‘When RNAs come full circle: new regulators of healing after myocardial infarction’, by C. Zaragoza, https://doi.org/10.1093/eurheartj/ehag038.**

Translational Perspective Current MI treatments primarily focus on symptom relief while overlooking the underlying disease mechanisms, underscoring the need for targeted interventions. Macrophages, which play a crucial role in post-MI healing, have emerged as promising therapeutic targets; however, the role of circRNAs in macrophage-driven inflammation remains unclear.

In this study, the authors identify circHIPK2 as a key regulatory molecule, highly expressed in cardiac tissues from heart failure patients and associated with inflammatory gene signatures. In a murine MI model, circHIPK2 upregulation was found to promote macrophage pro-inflammatory polarization. Macrophage-specific inhibition of circHIPK2 alleviated adverse remodelling, inflammation, and fibrosis. These findings highlight circRNA-based macrophage modulation as a novel therapeutic strategy for treating cardiac dysfunction and other inflammatory diseases.

## Introduction

Heart failure (HF) following ischaemic events such as myocardial infarction (MI) remains a leading cause of death worldwide. Despite advancements in treatment modalities, including percutaneous coronary intervention (PCI), coronary artery bypass grafting (CABG), and pharmacological interventions targeting neurohumoral activation such as beta-blockers and angiotensin-converting enzyme (ACE) inhibitors, HF-related mortality remains high, especially within the 5 years post-diagnosis.^1,2^ Current therapies for MI are limited in their ability to reverse cardiac damage and often lack cell specificity, leading to undesirable side effects. These limitations highlight the urgent need for targeted therapies based on deeper mechanistic insights into MI pathophysiology.

Recent research has focused on two primary therapeutic strategies: (i) remuscularization of lost cardiomyocytes and (ii) enhancement of improved cardiac healing.^3–5^ While these approaches have shown promise in preclinical studies, their clinical translation has been hindered by persistent inflammation, often driven by necrotic cardiomyocytes.^6–8^ In this context, macrophages have emerged as a therapeutically addressable target in several cardiac diseases including MI.^9^ As the predominant immune cells infiltrating the injured heart, their shift from a pro-inflammatory to an anti-inflammatory phenotype is crucial for inflammation resolution, tissue homeostasis, and remodelling. During MI progression, this phenotypic switch is often impaired or delayed, leading to prolonged inflammation, excessive fibrosis, and cardiac dysfunction. Consequently, therapeutic modulation of macrophages represents a promising strategy to control inflammation and fibrosis for optimal post-MI remodelling.^10^

Emerging evidence highlights the critical role of transcriptional remodelling in the progression of HF, underscoring the potential of targeting stress-induced gene expression.^11^ Among these transcriptional changes, non-coding RNAs (ncRNAs) have gained attention for their regulatory roles in disease pathogenesis. NcRNAs are emerging as versatile regulatory molecules and therapeutic targets, originating not only from non-coding regions but also from introns, exons, or non-coding isoforms of protein-coding genes.^12^ A unique subclass of ncRNAs, circular RNAs (circRNAs), formed through back-splicing of precursor mRNA (pre-mRNA) that covalently links their 3′ and 5′ ends, offers distinct advantages for RNA-based therapies.^13–15^ Their circular structure confers resistance to exonuclease-mediated degradation, and their conservation and tissue-specific expression make them promising disease biomarkers and therapeutic targets.^16,17^ Recent efforts have emphasized rigorous validation of circRNAs, including confirmation of back-splice junctions (BSJs) and orthogonal detection methods, to ensure accurate quantification and reliable functional interpretation.^18^ Recent evidence links circRNAs to macrophage activation and polarization, identifying key roles in inflammatory conditions.^19^ For instance, circRasGEF1B stabilizes Intercellular Adhesion Molecule 1 (ICAM1) during pro-inflammatory macrophage,^20^ while circRNA_17725 attenuates arthritis by promoting anti-inflammatory macrophage polarization.^21^ These findings suggest macrophage-specific circRNAs could serve as therapeutic targets in inflammatory diseases, including MI. However, the role of circRNAs in cardiac macrophages dynamics during MI remains poorly understood.

In this study, we identified circHIPK2, a circRNA derived from the second exon of homeodomain-interacting protein kinase 2, as a cardiac macrophage-specific circRNA upregulated following MI. We further investigated its role in macrophage polarization and impact on post-MI remodelling through both *in vivo* and *ex vivo* platforms. Our findings provide novel therapeutic potential of macrophage-targeting strategies via circRNA modulation for improved cardiac remodelling following MI.

## Methods

Detailed experimental materials and methods are available in Supplementary materials online.

## Results

### Identification of macrophage-enriched circRNAs in heart failure and circHIPK2 expression is associated with macrophages polarization

**Figure 1:**
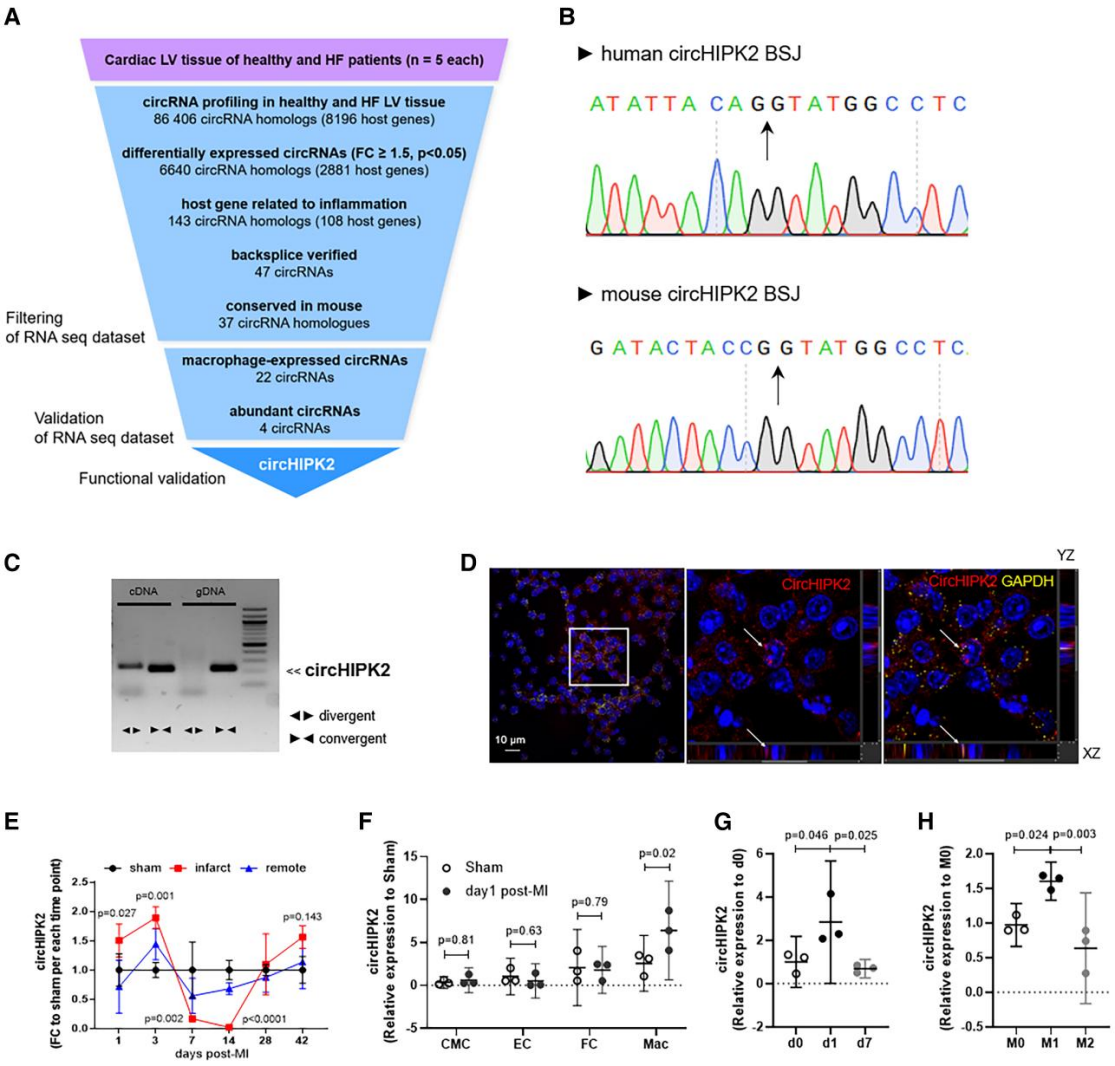
Macrophage-enriched circHIPK2 was identified from failing heart and validated in activated macrophages. (*A*) Schematic representation of the RNA-Seq data analysis pipeline for candidate selection. (*B*) Sanger sequencing chromatograms of circHIPK2 PCR products amplified from human and mouse cDNA; black arrow indicates the back-splice junction (BSJ). (*C*) Agarose gel electrophoresis of PCR products amplified using circHIPK2 primer (divergent) and HIPK2 primer (convergent) from cDNA or gDNA templates. (*D*) RNA fluorescence *in situ* hybridization (RNA-FISH) of circHIPK2 with a well-established cytoplasmic marker, GAPDH probe in Raw264.7 macrophages (scale bar, 10 μm). DAPI visualized nuclei. White arrowheads indicate cytoplasmic localization of circHIPK2 as observed in RNA-FISH experiment. (*E*) RT-qPCR analysis of circHIPK2 expression in myocardial tissues, sham, both infarct, and remote post-MI. Values are normalized to the sham group at each time point. (*F*) Cellular expression profile of circHIPK2 in major cardiac cell types (CMC, cardiomyocytes; EC, endothelial cells; FC, fibroblasts; Mac, macrophages) isolated 1 day post-MI (*n* = 3). (*G*) Time-course analysis of circHIPK2 expression in primary cardiac macrophages isolated at Day 0 (no infarct), Day 1, and Day 7 post-MI (*n* = 3). (*H*) Expression levels of circHIPK2 in unpolarized (M0) and polarized (M1: LPS-induced; M2: IL-4-induced) macrophages (*n* = 3). All data are shown as mean ± 95% CI. Statistical significance was determined using two-way ANOVA and Tukey’s multiple comparison test (*E* and *F*) or one-way ANOVA and Tukey’s multiple comparison test (*G* and *H*). Exact *P*-values are shown, and significance was defined as *P* < .05

To identify circRNAs involved in HF pathogenesis, we conducted global circRNA profiling in left ventricular tissue from healthy controls and HF patients (*n* = 5/group). Among 86 406 circRNAs analysed, 6640 exhibited significant differential expression (adj. *P* ≤ .05, FC ≥ 1.5). Focusing on inflammation-associated candidates, we filtered circRNAs derived from host genes linked to inflammatory pathways, yielding 143 circRNAs (108 annotated). *In silico* BSJs validation narrowed this to 47 candidates. Subsequent prioritization based on evolutionary conservation, macrophage expression, and abundance identified four candidates: circPDPK1, circZKSCAN1, circLrch3, and circHIPK2. Experimental validation highlighted circHIPK2 (hsa_circ_0001756/mmu_circ_0001468) as the most promising candidate for inflammation–HF interplay (*Figure 1A*).

CircHIPK2 exhibited high expression in HF patient-derived cardiac tissue and LPS-activated macrophages (see Supplementary data online, *Figure S1A* and *B*), positively correlating with inflammatory markers in failing hearts (see Supplementary data online, *Figure S1C* and *D*). Cross-species analysis revealed high sequence homology (human-mouse: 91.87%; rat-mouse: 98.45%; human-pig: 90.53%) (see Supplementary data online, *Figure S1E*). Divergent primer-based qPCR confirmed circHIPK2’s back-splicing in human/mouse samples, validated by Sanger sequencing and agarose gel electrophoresis (*Figure 1B* and *C*, Supplementary data online, *Table S1*). Subcellular fractionation and RNA fluorescence *in situ* hybridization (RNA-FISH) experiments demonstrated predominantly cytoplasmic localization of circHIPK2 (*Figure 1D*, Supplementary data online, *Figure S1F*). Treatment with transcription inhibitor actinomycin D demonstrated a longer half-life for circHIPK2 compared with its linear counterpart, HIPK2 (see Supplementary data online, *Figure S1G*). These findings establish circHIPK2 as a conserved, stable, and cytoplasmic circRNA with potential significance in the context of inflammation and heart failure.

In a mouse MI model, the tissue expression of circHIPK2 paralleled inflammatory dynamics^22^: elevated during early pro-inflammatory phases (days 1–3 post-MI) and reduced during later reparative stages (weeks-months) (*Figure 1E*, Supplementary data online, *Figure S2A*). A similar temporal pattern was observed in a mouse ischaemia/reperfusion (I/R) model (see Supplementary data online, Figure  *S2B*). In contrast, no significant change in circHIPK2 expression was detected in the transverse aortic constriction (TAC) model (see Supplementary data online, Figure  *S2C*). To assess the clinical relevance of these findings, we examined circHIPK2 expression in human heart tissue from patients with ischaemic cardiomyopathy (ICM) vs healthy controls (see Supplementary data online, Figure  *S2D*, Supplementary data online, *Table S2*). Consistent with the results from the mouse ischaemic models, circHIPK2 expression was elevated in ICM tissue relative to healthy myocardium. Next to determine its cellular source, cardiac fractionation was performed using harvested LV tissue 1 day post-MI, when circHIPK2 expression was markedly elevated. In sham-operated hearts, circHIPK2 was higher in fibroblast and macrophage fractions vs cardiomyocytes or endothelial cells. However, following MI, circHIPK2 expression was significantly increased only in macrophage fractions on Day 1 post-MI (*Figure 1F*). To further investigate whether circHIPK2 expression is associated with macrophage polarization states, we measured circHIPK2 levels in cardiac macrophages isolated on Day 1 post-MI (pro-inflammatory phase), Day 7 post-MI (anti-inflammatory/reparative phase), and from non-MI controls (Day 0). Interestingly, circHIPK2 expression was specifically upregulated in pro-inflammatory macrophages on Day 1 post-MI compared with control macrophages (Day 0) (*Figure 1G*). Consistent with these findings, our *in vitro* experiments demonstrated that LPS-stimulated pro-inflammatory macrophages exhibited significantly increased circHIPK2 expression (*Figure 1H*). Conversely, circHIPK2 levels markedly decreased in reparative macrophages (Day 7 post-MI) and in IL-4-induced anti-inflammatory (M2) macrophages (*Figure 1G* and *H*). These findings suggest that circHIPK2 plays a pivotal role in MI-related inflammatory response, particularly by influencing macrophage polarization.

### CircHIPK2 inhibition promotes anti-inflammatory macrophage polarization and modulates secretory factors influencing neighbouring cardiac cells

**Figure 2:**
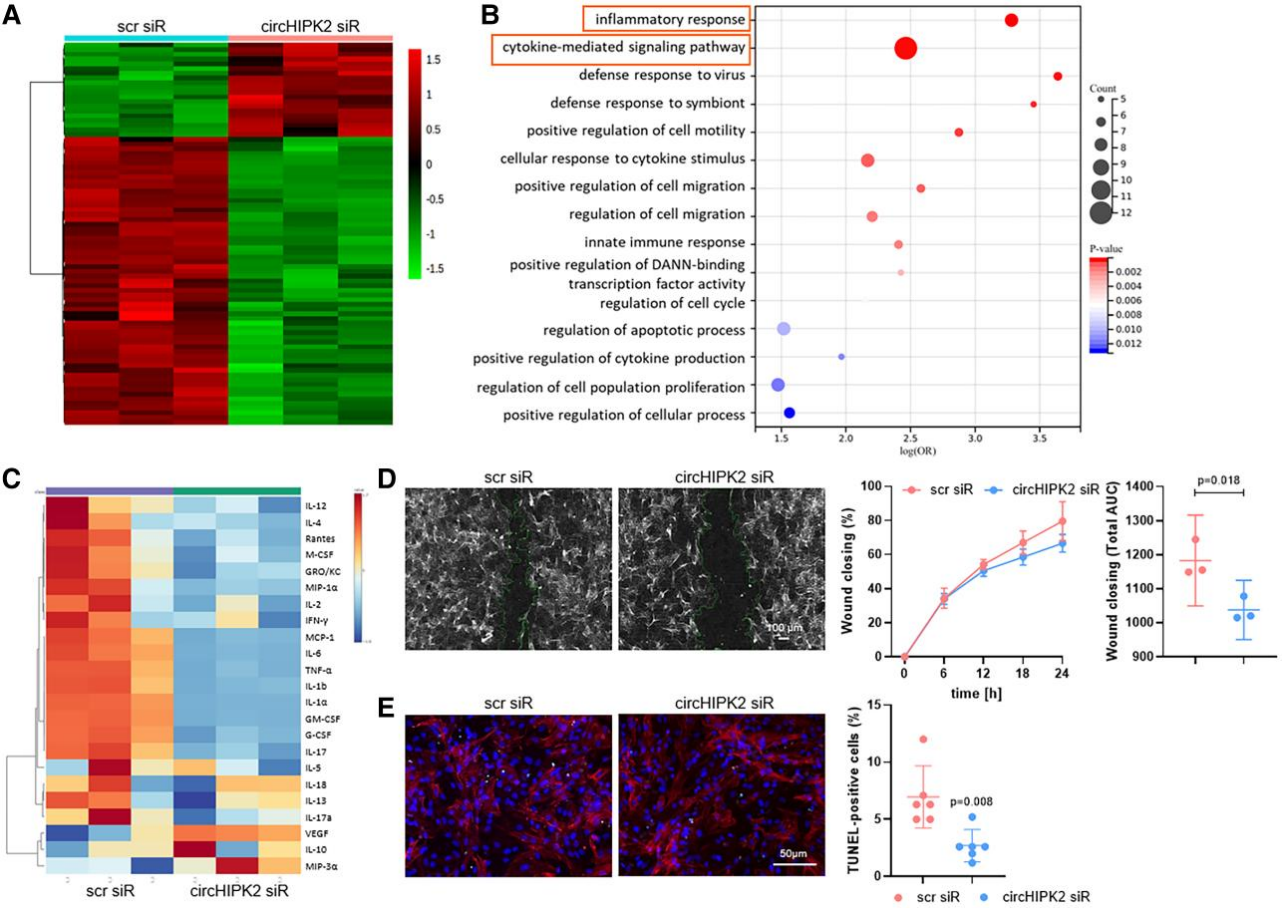
Knockdown of circHIPK2 attenuated inflammatory signalling and mediators in RNA-Seq and secretome analyses. (*A*) Heatmap depicting RNA-Seq data for dysregulated genes in Raw264.7 cells treated with circHIPK2 siRNA compared with scramble siRNA. The colour key at the top right corner represents log fold change (logFC) values. (*B*) KEGG analysis of the dysregulated gene pathways from RNA-Seq data. Odds ratios were derived from Enrichr based on OR = (a/b)/(c/d), where *a–d* represent the distribution of input genes relative to each gene set; larger OR values denote higher enrichment. The orange box in the bubble plot highlights the most prominently regulated cellular processes after circHIPK2 knockdown, emphasizing inflammatory and cytokine-mediated signalling pathways. (*C*) Heatmap illustrating the cytokine profile of secretome collected from bone marrow derived macrophages cells treated with circHIPK2 siRNA compared with scramble siRNA. Conditioned medium (secretome) collected from no infarct (Day 0 post-MI) cardiac macrophages treated with either circHIPK2 siRNA or scramble siRNA was used to culture (*D*) cardiac fibroblasts (CFs) isolated from Day 1 post-MI mouse hearts. CFs in wound closure assay, and (*E*) HL-1 mouse cardiomyocyte cell line in TUNEL assay. (*D*) Representative images (left panel) of wound closing of CFs were taken after 48 h. The wound area has been measured every 6 h and plotted (right panel) and the overall migratory response was quantified by area-under-the-curve (AUC) analysis (*n* = 3). For TUNEL assay (left panel), Alexa 594: α-actinin (cardiomyocytes marker); Alexa 488: TUNEL-positive nucleus (apoptotic cells); DAPI: nucleus. Quantification of TUNEL-positive cells (right panel) in experimental groups (*n* = 4). All data are presented as individual values (scatter) with mean ± 95% CI. Statistical significance was defined as *P* < .05; exact *P*-values are indicated

To gain further functional insights, we employed siRNA specifically targeting the unique BSJ of circHIPK2 (see Supplementary data online, *Figure S3A*, Supplementary data online, *Table S3*), achieving selective suppression of circHIPK2 while preserving linear HIPK2 transcript levels (see Supplementary data online, *Figure S3B*). To investigate the potential role of circHIPK2 downregulation in modulating inflammatory response under pro-inflammatory polarization conditions, mimicking the inflammatory state observed in the early stages post-MI, circHIPK2-silenced macrophages were subjected to LPS stimulation. Notably, circHIPK2 knockdown was sustained post-LPS stimulation (see Supplementary data online, *Figure S3B*). Subsequently, RNA sequencing (RNA-Seq) revealed that circHIPK2 inhibition induced transcriptional changes in genes mainly associated with inflammatory pathways and cytokine-mediated signalling, underscoring a strong link between circHIPK2 expression and inflammation (*Figure 2A* and *B*). The inhibitory effects of circHIPK2 knockdown (KD) on key inflammatory genes (e.g. TNF-α, CCL7, and IL-1b) were further validated in mouse bone marrow-derived macrophages (mBMDM) following LPS stimulation (see Supplementary data online, *Figure S3C*). Oligonucleotides sequences are listed in Supplementary data online, *Table S1*. Additionally, silencing circHIPK2 significantly reduced reactive oxygen species (ROS) production, shown by decreased DCFDA fluorescence (see Supplementary data online, *Figure S3D*). To next investigate gain-of-function, we constructed a circHIPK2 overexpression cassette comprising the circHIPK2 exon flanked by intronic sequences containing circularization Alu elements (see Supplementary data online, *Figure S3E*). The overexpression construct was subcloned into a pcDNA backbone. The specificity of circHIPK2 overexpression without altering its host transcript (linear HIPK2) was confirmed by RT-qPCR and agarose gel electrophoresis (see Supplementary data online, *Figure S3F*). Overexpression in mBMDM elevated inflammatory gene expression (TNF-α, CCL7, IL-1β) (see Supplementary data online, *Figure S3G*) and ROS generation (see Supplementary data online, *Figure S3H*). These findings suggest that selective inhibition of circHIPK2 in macrophages may play a crucial role in modulating the pro-inflammatory activation of macrophages and the subsequent inflammatory response. To further investigate the secretory factors of macrophages and their interactions within the cardiac cellular network, we applied secretome analysis and co-culture experiments with other cardiac cell types (*Figure 2C–E*). Remarkably, circHIPK2 KD suppressed significantly reduced the secretion of pro-inflammatory cytokines such as IL-1b, TNF-α, and MCP-1 (*Figure 2C*, Supplementary data online, *Table S4*). Intriguingly, conditioned medium collected from circHIPK2-silenced macrophages markedly diminished cardiac fibroblast migration and inhibited cardiomyocyte apoptosis (*Figure 2D* and *E*). These findings suggest that targeting circHIPK2 specifically in macrophages holds potential as a novel therapeutic strategy to modulate the post-MI inflammatory environment in the myocardium.

### Design and efficacy of macrophage-targeted circHIPK2 inhibition in a mouse model of MI

To translate our *in vitro* findings to *in vivo*, we developed an adeno-associated virus (AAV) vector system for macrophage-specific targeting of circHIPK2.^23–25^ The construct incorporated a CD68 promoter for macrophage specificity, a GFP tag for tracking, and a short hairpin RNA (shRNA) targeting circHIPK2 or a scramble sequence (see Supplementary data online, *Figure S4A*). AAV6 was used in *in vitro* validation for its macrophage tropism. The AAV6-circHIPK2 shRNA effectively knocked down circHIPK2 expression specifically in macrophages, without off-target effects in fibroblasts or cardiomyocytes (see Supplementary data online, *Figure S4B*). For *in vivo* applications, we utilized AAV9, which offers superior cardiac gene transfer efficiency. Next, to examine the target efficiency of the AAV9 virus in a disease context, adult male mice (8–10 weeks old) received 1 × 10^12^ viral genomes (vg) of AAV9-circHIPK2-shR or AAV9-scramble-shR (scr-shR). Seven days post-injection, mice underwent left anterior descending ligation to induce MI (see Supplementary data online, *Figure S4C*). Hearts were harvested 7 days post-MI for cardiac cell isolation. Additionally, macrophages isolated from spleen, a well-established macrophage reservoir, served as controls. The GFP signals from the injected viruses remained detectable in infarct regions at 14 days post-injection, but not in remote regions, confirming localized viral expression (see Supplementary data online, *Figure S4D*). Fluorescence-activated cell sorting analysis using GFP and CD68 markers revealed four subpopulations: GFP^+^CD68^+^ (AAV-transduced macrophages), GFP^−^CD68^+^ (non-transduced macrophages), GFP^+^CD68^−^ (AAV-transduced non-macrophage cells), and GFP^−^CD68^−^ (potentially dead cardiomyocytes or fibroblasts) (see Supplementary data online, *Figure S4E* for heart and *S4F* for spleen). AAV9-circHIPK2-shR significantly and selectively inhibited circHIPK2 expression in GFP^+^CD68^+^ populations from both heart and spleen, with no inhibitory effects in non-macrophage cell populations (GFP^−^CD68^+^, GFP^−^CD68^−^) (see Supplementary data online, *Figure S4E* and *F*). Notably, inflammatory gene expression was reduced in macrophages from AAV9-circHIPK2-shR-injected mice compared with controls (scr-shR), verifying our *in vitro* findings (see Supplementary data online, *Figure S4G* and *H*). In conclusion, we have successfully established an AAV9-mediated macrophage-specific KD system for circHIPK2 *in vivo*, providing a robust platform for further investigation of circHIPK2’s role in MI.

### Inhibition of macrophage-specific circHIPK2 improves cardiac function

**Figure 3:**
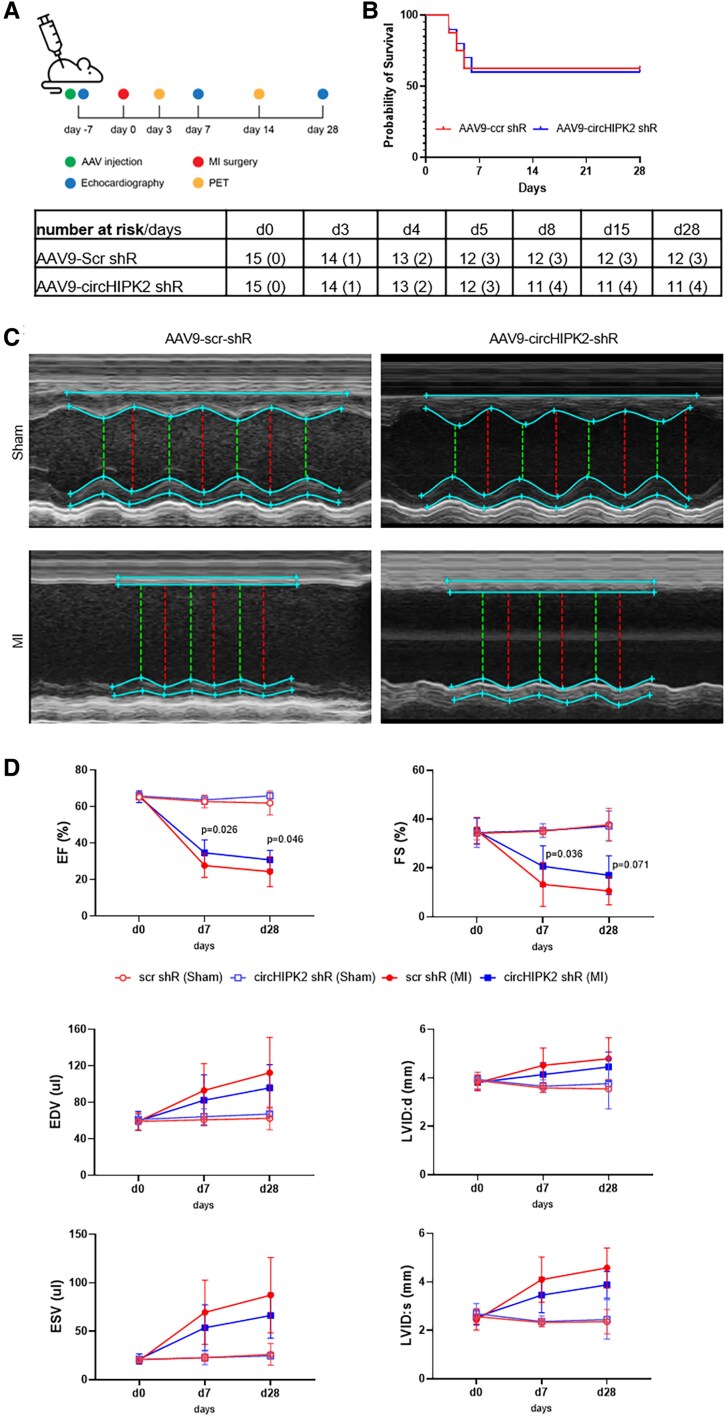
Macrophage-specific inhibition of circHIPK2 protected left ventricular dysfunction. (*A*) Scheme of animal study timeline. Mice were administered with macrophage-targeting AAV9 virus via intravenous injection. Seven days after injection, these mice were further subjected to coronary artery ligation to induce myocardial infraction (MI). Echocardiographic analysis was performed at Days 0 (pre-infarction), 7, and 28 post-MI. Mice were sacrificed on Day 28 post-MI for further analysis. (*B*) Kaplan–Meier survival curves of mice post-MI treated with AAV9-scr-shR or AAV9-circHIPK2-shR. Time 0 represents the day of MI surgery, and survival was monitored through Day 28 post-MI. No significant difference (*P* = .97) in survival was observed between groups. Numbers at risk are shown below the survival curves. (*C*) Representative left ventricular (LV) M-mode long-axis echocardiography images at day 28 post-MI and sham in AAV9-scr shR and AAV9-circHIPK2 shR injected mice. (*D*) Echocardiographic assessment of cardiac function parameters in MI and sham mice, including left ventricular ejection fraction (LVEF), fractional shortening (FS), LV volumes (end-diastolic volume [EDV], end-systolic volume [ESV]), and LV dimensions (LV internal diameter at diastole [LVID:d] and systole [LVID:s]). Data are shown as means ± SD (*n* = 7–8 mice per group). Statistical significance was determined using multiple unpaired *t*-tests comparing the AAV9-circHIPK2 shR group to the AAV9-scr shR group at each time point. Exact *P*-values are shown when *P* < .10

Next, to investigate the therapeutic potential of macrophage-specific circHIPK2 inhibition *in vivo*, we employed a well-established mouse model of MI. As illustrated in *Figure 3A*, mice were injected intravenously with AAV9-scr-shR or AAV9-circHIPK2-shR (MOI 1 × 10^12^ vg per mouse) on Day −7, followed by MI induction on Day 0 and sacrifice on Day 28. Cardiac function was assessed serially using echocardiography at Days 0, 7, and 28 post-MI. AAV9 treatment did not influence survival, rupture, or infarct size at d28 post-MI (*Figure 3B*, *S5A* and *B*). However, macrophage-specific circHIPK2 inhibition significantly improved cardiac function, as evidenced by enhanced ejection fraction (EF), fractional shortening (FS), LV volume, and LV dimensions (*Figure 3C, 3D*, Supplementary data online, *Table S5*). Body weight, LV mass, and spleen mass remained unchanged (see Supplementary data online, *Figure S5C*). Importantly, cardiac improvement was evident by d7 post-MI and persisted through d28. In the mouse MI model, the first week following MI is critical for early cardiac remodelling, encompassing processes such as inflammation resolution and fibroblast activation. Impaired or delayed early remodelling can result in prolonged inflammation and excessive fibrosis, ultimately contributing to cardiac dysfunction and HF. Our findings suggest circHIPK2 expression in cardiac macrophages plays a significant role in early post-MI remodelling.

### Inhibition of macrophage-specific circHIPK2 attenuates cardiac inflammation and fibrosis

**Figure 4:**
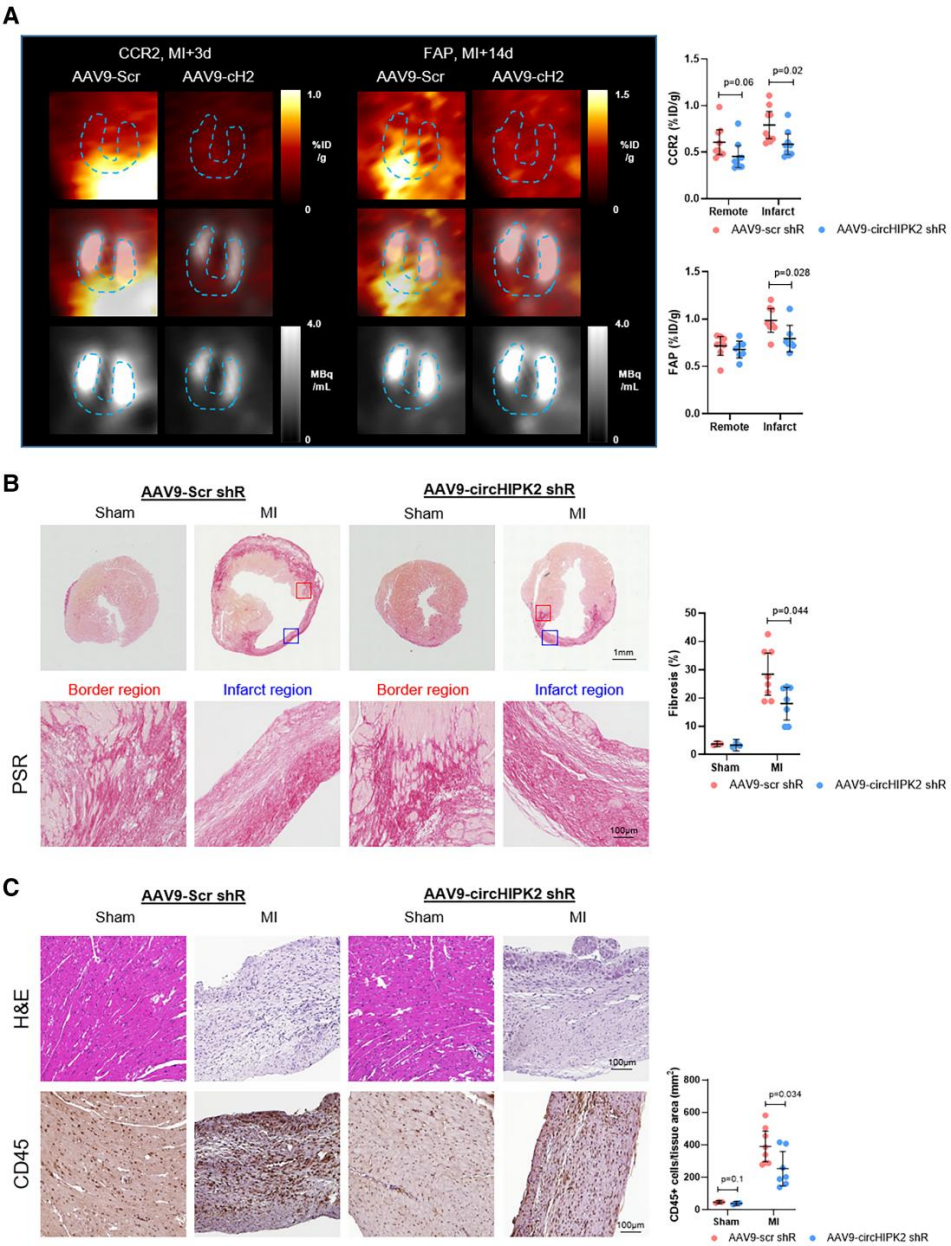
Macrophage-specific inhibition of circHIPK2 regulated immune cell infiltration and fibroblast activation post-MI. (*A*) Representative horizontal long axis positron emission tomography (PET) images of infarcted mouse hearts. CCR2^+^ inflammatory cell infiltration is visualized by ^68^Ga-ECL1i PET at 3 days post-MI (colour scale left in black box). FAP^+^ activated fibroblasts are detected by ^68^Ga-MHLL2 PET at 14 days post-MI (colour scale right in black box). Infarct region is identified by absence of ^18^F-2-fluorodeoxyglucose (FDG) uptake in the anterolateral wall (greyscale images). Data are expressed as per cent injected dose per gram of tissue (%ID/g), representing the mean voxel intensity (Bq/mL) within the region of interest normalized to the injected dose. (*B*) Picrosirius Red staining (PSR) for fibrosis assessment in heart tissue across four experimental groups (AAV9-scr-shR sham, AAV9-circHIPK2-shR sham, AAV9-scr-shR MI, AAV9-circHIPK2-shR MI) at 28 days post-MI or sham operation. Whole heart images (4× magnification) showing left ventricular fibrosis (left upper panel). Scale bar = 1 mm. Higher magnification (20×) of infarct and border regions (left lower panel). Scale bar = 100 μm. Quantification of collagen deposition per whole heart (right, bar graph). (*C*) Representative images of H&E staining (upper panel) and CD45 immunohistochemistry (bottom panel) of heart sections in each group. Scale bar = 100 μm. Quantitation of CD45+ inflammatory cells infiltration per tissue area (mm^2^) (right, bar graph). Data are presented as mean ± 95% CI (*n* = 7–8 per group). Statistical significance was assessed by performing multiple unpaired *t*-tests to compare the AAV9-circHIPK2 shR group with the AAV9-scr shR group at each of the sham and MI. (*A*–*C*). Exact *P*-values are shown for the indicated group comparisons

To further explore the role of circHIPK2 in early and mid-term cardiac remodelling, we employed molecular imaging using positron emission tomography (PET) to visualize C-C chemokine receptor type 2 (CCR2) and fibroblast activation protein (FAP). CCR2 marks infiltrating monocytes/macrophages linked to inflammation in failing hearts,^26^ while FAP indicates activated fibroblasts.^27,28^ PET imaging was conducted on a cohort of mice, targeting CCR2 at Day 3 (early remodelling) and FAP at Day 14 (mid-term remodelling) post-intervention, to evaluate the effects of AAV9-circHIPK2-shR on these distinct remodelling processes. Quantitative analysis of PET images revealed significantly higher tracer uptake in the infarct region compared with remote myocardium (*Figure 4A*). Notably, AAV9-circHIPK2-shR-injected group exhibited reduced CCR2 (Day 3) and FAP (Day 14) signals compared with AAV9-scr-shR control group (*Figure 4A*), with no significant differences in remote myocardial regions between the groups. These findings underscore the substantial involvement of macrophages in cardiac remodelling and suggest that macrophage-specific circHIPK2 inhibition mitigates inflammation and fibroblast activation. To further explore the impact on continued late-stage cardiac remodelling, histological analyses were performed to assess collagen deposition and inflammatory cell infiltration. Picrosirius Red (PSR) staining confirmed reduced fibrosis in treated mice compared with controls, with no difference in sham mice (*Figure 4B*). Additionally, haematoxylin and eosin (H&E) staining and CD45 immunohistochemistry showed decreased inflammatory cell infiltration in the infarcted region of the AAV9-circHIPK2-shR-injected group compared with controls group (*Figure 4C*). Importantly, to further validate the effect of AAV9-circHIPK2-shR at an intermediate time point, we harvested hearts at Day 7 post-MI for additional analyses. At this time point, the AAV9-circHIPK2-shR group already displayed a marked reduction in inflammatory cell infiltration and notably diminished collagen deposition compared with controls (see Supplementary data online, *Figures S5D* and *E*). Moreover, consistent with the PET findings, the AAV9-circHIPK2-shR group showed lower levels of CCR2 and CCL2 gene expression, particularly in the infarct region at Day 7 post-MI (see Supplementary data online, *Figure S5F*). Together, these observations indicate that macrophage-specific inhibition of circHIPK2 attenuates early inflammation, suppresses subsequent fibroblast activation and collagen deposition, and ultimately mitigates late-stage remodelling following myocardial infarction.

### Inhibition of circHIPK2 lead to M2 polarization of macrophages resulting in improved cardiac remodelling

**Figure 5:**
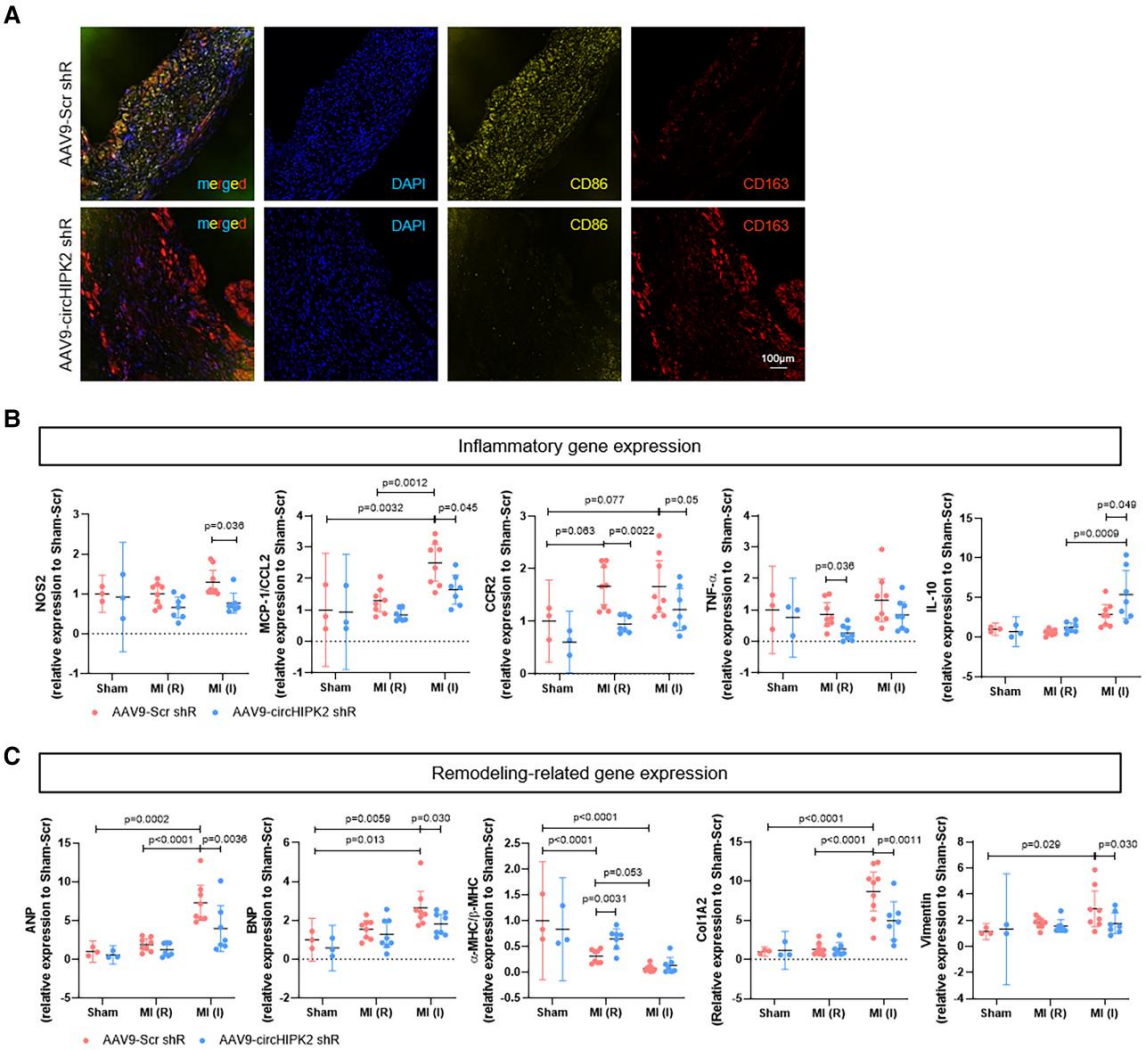
Macrophage-specific circHIPK2 inhibition promoted M2 macrophage polarization and attenuates adverse cardiac remodelling post-MI. (*A*) Representative immunofluorescence images of pro-inflammatory (CD86) and anti-inflammatory (CD163) macrophage markers in the infarct region of post-MI myocardium, comparing AAV-scr shR and AAV-circHIPK2 shR groups. Nuclei counterstained with DAPI. Scale bar = 100 μm. Images are representative of 6–10 fields of view from *n* = 7–8 biological replicates per group. (*B–E*) RT-qPCR analysis of myocardial gene expression related to (*B*) inflammation and (*C*) cardiac remodelling (hypertrophy and fibrosis) in sham, remote, and infarct zones of MI hearts, comparing AAV9-scr shR and AAV9-circHIPK2 shR groups. Data are presented as mean ± 95% CI (*n* = 7–8 mice per group). Exact *P*-values < .05 vs the indicated groups are shown. Statistical analyses were performed using two-way ANOVA followed by Tukey’s multiple-comparisons test

Next, immunohistochemical analyses were performed to assess the effect of circHIPK2 inhibition on cardiac macrophages in the infarct region post-MI. Macrophage polarization was evaluated by labelling pro-inflammatory macrophages with CD86 and anti-inflammatory macrophages with CD163. In AAV9-scr-shR hearts, CD86^+^ macrophages predominated, whereas significantly increased CD163^+^ macrophages were observed in the AAV9-circHIPK2-shR-hearts, consistent with *in vitro* findings that circHIPK2 inhibition promotes anti-inflammatory macrophage polarization (*Figure 5A*). Cardiac gene expression analysis demonstrated increased levels of pro- and anti-inflammatory markers in both infarct and remote zones post-MI vs sham, with more pronounced elevation in the infarct region. Notably, within the infarct region, AAV9-circHIPK2-shR administration significantly upregulated anti-inflammatory gene expression while downregulating pro-inflammatory genes compared with control group (*Figure 5B*). These results recaptured our *in vitro* findings that AAV9-circHIPK2-shR facilitates inflammatory resolution by promoting anti-inflammatory macrophage polarization. Additionally, MI-induced elevations in genes associated with cardiac hypertrophy (ANP, BNP, αMHC/βMHC) and fibrosis (Col1a2, Vimentin) were observed in both infarct and remote regions at 28 days post-MI. However, these increases were markedly attenuated in mice treated with AAV9-circHIPK2-shR. Importantly, AAV9-circHIPK2-shR alone did not alter the expression of these genes in sham mice (*Figure 5C*). Beyond its cardiac effects, AAV9-circHIPK2-shR also mitigated systemic inflammation, specifically, in macrophage-enriched organs such as the spleen and the kidney (see Supplementary data online, *Figure S6A*), and lowered creatine kinase activity (see Supplementary data online, *Figure S6B*). These findings highlight that AAV9-circHIPK2-shR not only alleviates cardiac inflammation and injury following MI but also exerts protective effects on systemic inflammation associated with myocardial damage.

### BRCA1 enhances circHIPK2 expression during the inflammatory response in macrophages

We employed ESEfinder 3.0 to investigate the upstream regulator of circHIPK2, especially enhancer regions that interact within the circularization ALU elements (see Supplementary data online, *Figure S7A*, Supplementary data online, *Table S6*). Notably, Brca1 expression was upregulated in inflammatory macrophages upon LPS stimulation, coinciding with increased circHIPK2 levels (see Supplementary data online, *Figure S7B*). We then performed siRNA-mediated KD of selected candidate factors, and results showed that Brca1 emerged as the most promising regulator as its inhibition led to a significant decrease in circHIPK2 expression, but not HIPK2 (see Supplementary data online, *Figure S7C* and *D*). These findings suggest that Brca1 regulates circHIPK2 expression through a mechanism independent of the host gene. Similarly, co-transfection of a circHIPK2 overexpression plasmid with Brca1 siRNAs significantly reduced circHIPK2 levels in macrophages compared with co-transfection with control siRNAs, indicating that Brca1 promotes circHIPK2 biogenesis (see Supplementary data online, *Figure S7E*).

### CircHIPK2 physically interacts with G3BP1 to regulate inflammatory response in macrophages

**Figure 6:**
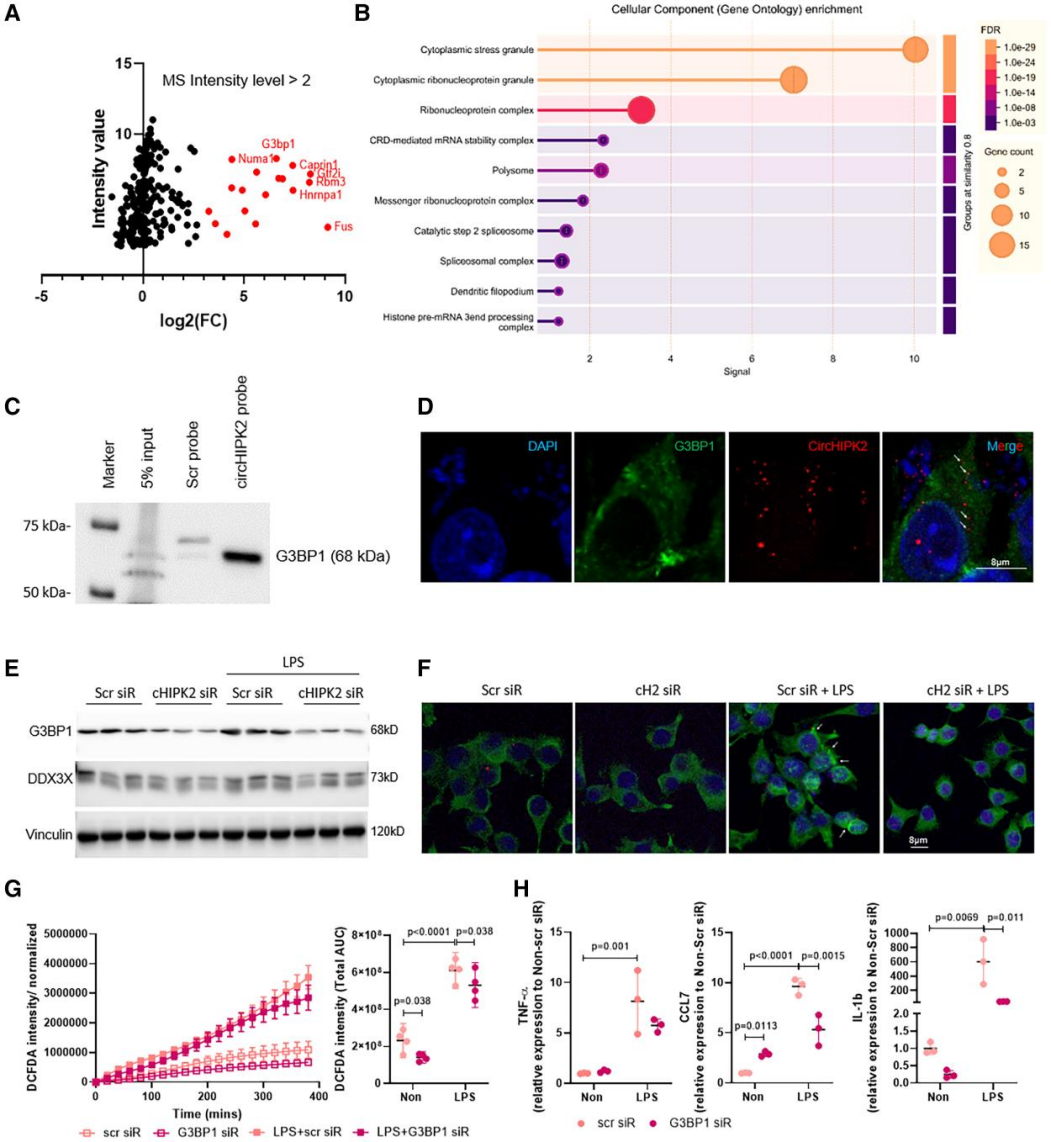
CircHIPK2 modulated stress granule (SG) formation through interaction with RNA-binding protein G3BP1. (*A*) MA plot illustrating the relationship between intensity values and fold changes (log2FC) of proteins. Red dots indicate proteins significantly enriched by the circHIPK2 probe compared with the scr probe. (*B*) Gene ontology (GO) analysis of 18 proteins enriched by circHIPK2 probe, predominantly associated with cytosolic stress granule formation. (*C*) Western blot analysis of G3BP1 protein levels in Raw264.7 macrophages following circHIPK2 and scrambled RNA pulldown using biotin-labeled DNA probes. (*D*) RNA fluorescence *in situ* hybridization (RNA-FISH) of circHIPK2 (red) co-stained with G3BP1 (green) and DAPI (blue) in Raw264.7 macrophages. Scale bar = 8 μm. Representative images from three independent experiments. (*E*) Western blot analysis demonstrating the effect of circHIPK2 inhibition on the expression of G3BP1 protein, stress granule-related protein DDX3X, and vinculin. (*F*) Immunofluorescence microscopy analysis of cytoplasmic stress granule formation, as visualized by G3BP1 protein localization (green fluorescence), in Raw264.7 macrophages following LPS stimulation and circHIPK2 inhibition. (White arrows indicate areas of stress granule condensation). Scale bar = 8 μm. Representative images from three independent experiments. (*G*) Cellular ROS production was measured in circHIPK2-overexpressed Raw264.7 macrophages upon inhibition of G3BP1. DCFDA fluorescence intensity was measured every 20 min for 6 h (left). The overall response was quantified as the area-under-the-curve (AUC, right). Data are shown as mean ± 95% CI (*n* = 4). *P*-values were determined by two-way ANOVA followed by Tukey’s multiple-comparisons test. (*H*) Relative inflammatory gene expression (TNF-α, CCL7, and IL-1b) was analysed in mBMDM transfected with G3BP1 siRNA upon LPS stimulation. Data are shown as mean ± SD (*n* = 3). *P*-values were calculated using two-way ANOVA followed by Tukey’s multiple-comparisons test. Exact *P*-values are provided for the indicated comparisons

Next, to investigate mechanism underlying circHIPK2-mediated anti-inflammatory effects, we conducted RNA pulldown using biotin-labelled probes targeting the BSJ of circHIPK2 or a scramble sequence, followed by mass spectrometry analysis (see Supplementary data online, *Figure S8A*). This approach revealed 18 proteins consistently enriched in circHIPK2 probe samples across three independent pulldown sets (*Figure 6A*, Supplementary data online, *Table S7*). Subsequent STRING analysis highlighted Gene Ontology (GO) terms enrichment related to cytoplasmic stress granules (SG) and ribonucleoprotein granules (*Figure 6B*). Among top candidates, G3BP1 was identified as the predominant binding partner of circHIPK2 (*Figure 6C*, Supplementary data online, *Figure S8B*). This specific interaction was subsequently validated through immuno-RNA-FISH colocalization and RNA immunoprecipitation (RIP) assays (*Figure 6D*, Supplementary data online, *Figure S8C*). Next, we conducted western blot analysis to explore the mechanism underlying the interaction between circHIPK2 and G3BP1, a core protein for SG formation under conditions such as viral infection or oxidative stress. Following LPS treatment, the protein levels of SG components, including G3BP1 and DDX3X, were significantly elevated (*Figure 6E*), while inhibition of circHIPK2 markedly reduced these increases. Immunofluorescence imaging confirmed enhanced SG formation during LPS stimulation, marked by G3BP1 localization, was impaired upon circHIPK2 downregulation (*Figure 6F*). Conversely, circHIPK2 overexpression increased G3BP1 protein level and enhanced SG formation, whereas G3BP1 inhibition disrupted SG stability (see Supplementary data online, *Figure S8D*, *Figure S6E*).

Consistently, we observed that G3BP1 inhibition significantly attenuated LPS-induced ROS production and inflammatory gene expression (*Figure 6G* and *H*), mirroring circHIPK2 inhibition effects. These findings indicate that inflammatory attenuation observed upon circHIPK2 inhibition is partially attributable to modulating G3BP1 activity. Supporting this, inflammatory gene expression induced by circHIPK2 overexpression was reversed by G3BP1 inhibition (see Supplementary data online, *Figure S8F*). Similarly, Day 1 post-MI macrophages exhibited elevated G3BP1 levels and inflammatory gene expression (CCL7, TNF-α, and IL-1α) compared with Day 0 non-infarcted and Day 7 reparative macrophages (see Supplementary data online, *Figure S8G*). These results demonstrate circHIPK2 synergizes with G3BP1 to promote SG formation and inflammatory cascades under stress conditions. Our *in vivo* studies further validated these findings. Mice treated with AAV9-circHIPK2 shRNA exhibited markedly decreased G3BP1 expression in infarcted region, compared with AAV9-scr control group (see Supplementary data online, *Figure S8H*), aligning with *in vitro* findings. This indicates that macrophage-specific circHIPK2 inhibition mitigates post-MI inflammation by modulating G3BP1 and SG dynamics. Specifically, circHIPK2 inhibition disrupts its interaction with G3BP1, driving macrophage polarization towards an anti-inflammatory phenotype and facilitating the resolution of inflammation. We further mapped the G3BP1-binding region of circHIPK2. CatRAPID prediction revealed three potential sites: nucleotides 100–200, 200–400, and 800–900 bp (see Supplementary data online, *Figure S8I*). To validate these predictions, we generated deletion mutants lacking each of these regions and conducted RNA pulldown. Notably, deletion of the 800–900 bp region significantly reduced the binding affinity to G3BP1, suggesting that this segment represents the primary interaction site between circHIPK2 and G3BP1 (see Supplementary data online, *Figure S8J*).

### NF-κB links G3BP1-mediated SG formation to the inflammatory response

Next, to determine whether SG formation itself modulates macrophage polarization independently of circHIPK2, we treated macrophages with sodium arsenite, a well-established SG inducer. Arsenite stimulation did not alter circHIPK2 or its host gene HIPK2 expression (see Supplementary data online, *Figure S9A*), suggesting SG formation may occur through both circHIPK2-dependent and circHIPK2-independent pathways. Functionally, arsenite-treated macrophages exhibited significantly increased pro-inflammatory gene expression (TNF-α, CCL7, IL-6) and ROS production, while reduced anti-inflammatory markers (Arg-1, IL-10) (see Supplementary data online, *Figure S9B*–*D*). To understand downstream effects of SG formation, we focused on NF-κB, a known mediator of inflammatory responses. Based on the study by Reineke *et al*.^29^ G3BP1-induced SG activated innate immune pathways via NF-κB and JNK signalling. Consistent with these findings, luciferase reporter assays demonstrated arsenite treatment significantly increased NF-κB activity (see Supplementary data online, *Figure S9E*). However, the shift towards an anti-inflammatory phenotype upon NF-κB inhibition further support a G3BP1–SG-mediated inflammatory response via NF-κB activation (see Supplementary data online, *Figure S9G*–*I*). Importantly, circHIPK2 KD suppressed NF-κB activation in LPS-stimulated macrophages, highlighting the role of the circHIPK2–G3BP1 axis in SG-driven inflammatory responses (see Supplementary data online, *Figure S9F*).

### Macrophage-targeting circHIPK2 modulation therapy showed translational potential in human patient-derived living myocardial slices

**Figure 7:**
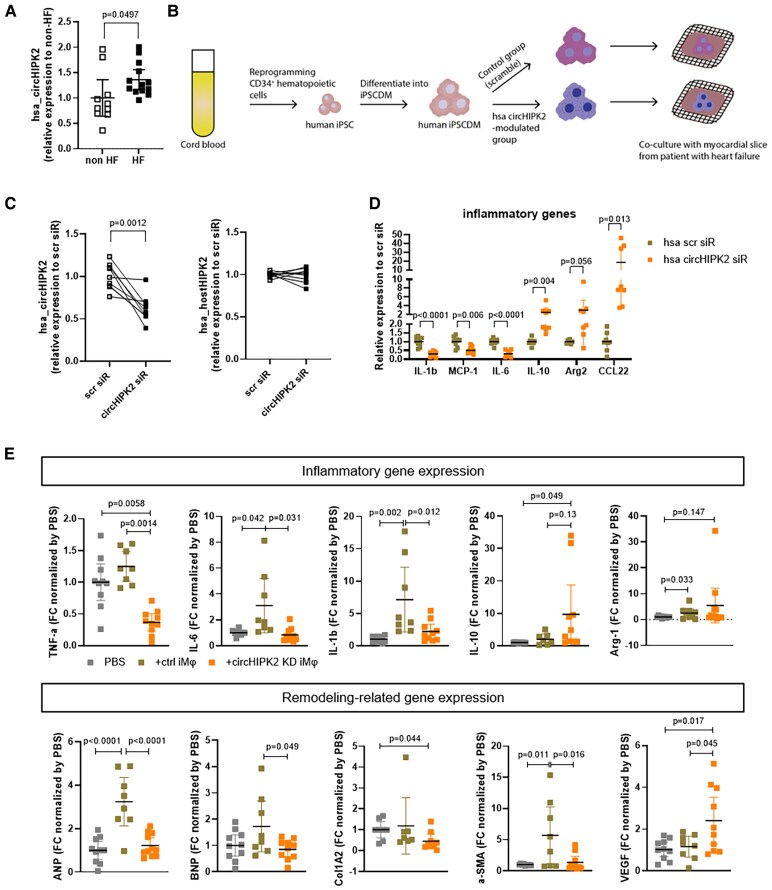
Inhibition of circHIPK2 in human iPSC-derived macrophages (iPSCDM) enhanced anti-inflammatory effects and improves cardiac remodelling in failing human hearts. (*A*) Elevated circHIPK2 expression in PBMC from heart failure patients compared with non-heart failure individuals (*n* = 10–13 per group). (*B*) Schematic representation co-culture experiment: circHIPK2-knockdown (KD) or control hiPSCDM on living myocardial slices (LMS) derived from patients with heart failure. (*C*) Specific suppression of circHIPK2 in hiPSCDM without altering host gene expression (*n* = 9). (*D*) circHIPK2 inhibition promotes anti-inflammatory macrophage polarization (*n* = 9). (*E*) RT-qPCR analysis of cardiac remodelling-related gene expression in LMS co-cultured with circHIPK2-KD or control hiPSCDM (+circHIPK2 KD iMϕ; +ctrl iMϕ), compared with PBS (macrophage-free) group. Inflammation-related genes (upper panel); hypertrophy and fibrosis-related genes (lower panel) (*n* = 5 patient-derived hearts, 1–2 slides per condition). Data are shown as means ± 95% CI. Statistical significance was determined using unpaired *t*-test (A and *C*) or two-way ANOVA followed by Tukey’s multiple comparisons test (D and *E*). Exact *P*-values are provided for the indicated comparisons

Next, to investigate the translational potential, we examined circHIPK2 function in human macrophages. Initially, we validated circHIPK2 expression in peripheral blood mononuclear cells (PBMCs) isolated from HF patients and non-HF controls. CircHIPK2 was significantly upregulated in HF-PBMC compared with non-HF (*Figure 7A*), corroborating our *in vitro* and *in vivo* findings. To evaluate translational efficacy, we inhibited circHIPK2 in human induced pluripotent stem cell-derived macrophages (hiPSCDM) without affecting its host gene, HIPK2 (*Figure 7B* and *C*). CircHIPK2 silencing significantly downregulated inflammatory gene expression in hiPSCDM (*Figure 7D*), consistent with earlier findings. The therapeutic potential of circHIPK2-KD macrophages was further assessed by co-culturing these cells with human living myocardial slices (LMS) (*Figure 7B*). LMS are ultrathin myocardial sections (100–400 μm, 7 × 7 mm dimensions) preserving multicellular architecture and physiology of cardiac tissue.^30^ Since infiltrated macrophages naturally deplete over time during LMS culturing, we replenished them by adding hiPSCDM. To simulate HF scenario, we co-cultured circHIPK2-inhibited or control-hiPSCDM on LMS-derived from a HF patient obtained during heart transplantation (*Figure 7B*). Detailed clinical information of patients is provided in Supplementary data online, *Table S8*. PBS served as a macrophage-free control group. Co-culturing with circHIPK2-KD hiPSCDM induced anti-inflammatory shift of myocardium environment, shown by reduced pro-inflammatory genes (TNF-α, IL-6, and IL-1b) and increased anti-inflammatory gene levels (IL-10 and Arg2) vs control macrophages and the PBS group (*Figure 7E*). No significant inflammatory differences were observed between control macrophages and PBS groups (*Figure 7E*). Co-culturing with control macrophages significantly elevated the expression of hypertrophy (ANP and BNP) and fibrosis (Col1a2 and α-SMA) genes compared with the PBS group, whereas circHIPK2-KD macrophages effectively reversed these increases, underscoring the critical role of macrophages in cardiac remodelling during HF progression (*Figure 7E*).

## Discussion

Our study demonstrates that circHIPK2 functions as a molecular switch in macrophages, playing a pivotal role in mitigating inflammation and decelerating MI progression. CircRNAs have emerged as key regulators in various pathological processes, with studies highlighting their involvement in macrophage-mediated inflammation. Among them, circCdr1as, mostly studied circRNA, promotes the anti-inflammatory phenotype of macrophages.^31^ However, circCdyl and circPrkcsh have been associated with pro-inflammatory macrophage activation and therapeutic potential in chronic inflammatory diseases.^32^ Additionally, circ_17725 promotes reparative macrophage polarization in arthritis models.^21^ However, the direct link between circRNAs and cardiac macrophages in the context of MI remains largely unexplored. Previous research revealed multifunctional roles of circHIPK2 in various pathologies.^33–35^ In the cardiovascular context, it facilitates phenotypic switching of vascular smooth muscle cells (VSMCs) in angiotensin II-induced hypertension.^36^ Yet, function and mechanisms of circHIPK2 in MI, particularly within specific cellular contexts, remain poorly understood. Importantly, the induction of circHIPK2 in both permanent-ligation (*Figure 1E*) and ischaemia/reperfusion models (see Supplementary data online, *Figure S2B*) suggests its role in post-infarction inflammation may extend across clinically relevant injury contexts. While we selected the permanent occlusion MI model for mechanistic clarity, the parallel findings in the I/R model strengthen the translational relevance and warrant further investigation in reperfusion-based settings.

A key finding of our study is the association between circHIPK2 expression, macrophage polarization, and the subsequent post-MI inflammation. Our data demonstrate that circHIPK2 inhibition promotes anti-inflammatory macrophage polarization *in vitro*, in both rodent and human macrophages. Furthermore, macrophage-specific circHIPK2 inhibition via viral vector-mediated gene delivery demonstrated significant cardioprotection *in vivo* in a murine MI model. Among gene therapy systems, AAV vectors are favoured in cardiovascular application for their safety, low immunogenicity, and sustained and stable transgene expression.^37^ Innovations in capsid design and cell-specific promoters have improved AAV therapeutic efficiency. Ongoing clinical trials employing recombinant AAV (rAAV)-based therapies are underway for heart failure with reduced ejection fraction (HFrEF) using AAV1/SERCA2A, hypertrophic cardiomyopathy (HCM) using AAV9/MYBPC3, and other heart failure with AAV2/AAV8/I-1c.^38–40^ Despite significant progress, current AAV-based therapies primarily target cardiomyocytes, leaving macrophage-targeted approaches largely unexplored. To address this, we developed a macrophage-specific rAAV vector engineered with a CD68 promoter and shRNA against circHIPK2 or scrambled control. Based on prior reports of effective AAV9-shRNA-mediated gene silencing in the heart,^41,42^ we selected a dose of 1 × 10^12^ vg/mouse, administered 7 days before MI to align peak knockdown with post-MI inflammation onset. Our engineered vector achieved robust and specific circHIPK2 inhibition in macrophages, without off-target effects in non-macrophage populations (see Supplementary data online, *Figure S4B*, *E*). These results support the therapeutic potential of our strategy. Notably, systemic administration of our AAV system also reduced inflammatory gene expression in macrophage-rich organs such as the spleen and kidneys (see Supplementary data online, *Figure S6A*). Similarly, reduced PET signals of CCR2 and FAP in the liver were observed in AAV9-circHIPK2-shR administration compared with control group. While these effects may contribute to broader anti-inflammatory benefits, they also raise concerns about potential off-target effects in non-cardiac tissues. Importantly, circHIPK2 inhibition was well-tolerated up to 28 days post-MI, with no observable adverse effects on survival, body weight, cardiac mass, or injury markers (see Supplementary data online, *Figure S5A*–*C*). Although longer-term murine data are currently lacking, future studies in large animal models are necessary to evaluate the durability, safety, and translational potential of this approach. Additionally, future research should prioritize optimizing both organ and cell-type specificity to enhance the precision and therapeutic potential of macrophage-specific circHIPK2 inhibition.

The CCL2–CCR2 axis constitutes a well-established macrophage-mediated chemotactic signalling pathway, particularly in the context of myocardial injury and remodelling.^43,44^ In our study, both PET imaging and qPCR analyses demonstrated lower tissue expression levels of CCR2 and CCL2 in the AAV9-circHIPK2-shR group compared with the AAV9-scr-shR control at Days 7 and 28 post-MI. However, we acknowledge that specific monocyte and macrophage subsets in peripheral blood were not assessed in the present study. Future investigations incorporating longitudinal blood sampling and subset profiling (classical, intermediate, and non-classical monocytes) will be essential to delineate the systemic contribution of circHIPK2 to post-infarction inflammation.

CircRNAs exert their regulatory effects via interactions with cellular factors such as microRNAs and RNA-binding proteins.^14,45^ Pulldown coupled with STRING network analysis identified multiple candidates; from these, four proteins were prioritized for further validation: Ras-GTPase-activating protein binding protein 1 (G3BP1); Cell Cycle Associated Protein 1 (Caprin1); Heterogeneous Nuclear Ribonucleoprotein K (Hnrnpk), based on statistical significance and known SG associations; and Fused in sarcoma (FUS) based on highest fold enrichment and prior literature supporting potential interaction with circHIPK2.^46^ We identified a novel interaction between circHIPK2 and G3BP1, a scaffold protein essential for SG formation under stress. We demonstrate a robust association between G3BP1-mediated SG formation and pro-inflammatory macrophage polarization (see Supplementary data online, *Figure S9B*–*D*), consistent with previous reports implicating SGs in inflammatory signalling cascades.^29,47–49^ These findings indicate the circHIPK2-G3BP1 interaction is critical for SG persistence under stress conditions, while circHIPK2 inhibition disrupts SG assembly in macrophages.

Notably, G3BP1-mediated SGs have been observed in the coronary arteries of failing human hearts, suggesting a link between SG accumulation and advanced vascular pathology.^50^ In line with this, depletion of SG components like G3BP1 attenuates cytokine production and inflammatory signalling.^51–53^ Previous research suggested G3BP1-mediated SGs as therapeutic targets in vascular inflammation and atherosclerosis.^50,54^ Our findings support this and highlight the therapeutic relevance of targeting SGs in macrophages. However, it is important to note that direct inhibition of SG core components like G3BP1 may cause broad, unintended effects due to their pleiotropic cellular functions. In this context, circHIPK2 offers a more cell type–specific and physiologically tunable target, particularly given its enriched expression in cardiac macrophages. Our data suggest circHIPK2 inhibition modulates G3BP1 function and thereby attenuate SG formation, leading to reduced pro-inflammatory macrophage polarization and cardiac inflammation (*Structured Graphical Abstract*, *Figure 6E–H*). Importantly, the circHIPK2–G3BP1–SG axis also regulates NF-κB signalling (see Supplementary data online, *Figure S9G*–*I*), a central regulator of inflammation. While SG formation can occur independently of circHIPK2, its suppression significantly reduces NF-κB activity, suggesting its role in amplifying inflammatory signalling (see Supplementary data online, *Figure S9B*). Collectively, these findings establish circHIPK2 as a critical regulator of G3BP1-dependent SG formation and pro-inflammatory macrophage activation. Previous studies demonstrate circHIPK2’s diverse interactions in various contexts. In colorectal and colitis models, circHIPK2 binds EIF4A3 to enhance TAZ mRNA translation,^46^ while in myogenesis, it regulates proliferation via Rpl7.^55^ It also acts as a miR-506–3p sponge to modulate σ-1R in silicosis^56^; however, these interactions were not observed in our study. CircRNAs, unlike lncRNAs, exhibit high sequence conservation due to their derivation from precursor mRNAs.^57^ Our alignment revealed 91.97% sequence similarity for circHIPK2 between human and mouse (*Figure 1B*, Supplementary data online, *Figure S1C*). Exon 2 showed 90.53% conservation between human and pig, while only one nucleotide differed at the BSJ, underscoring circHIPK2’s evolutionary significance, particularly for large animal studies. Functionally, circHIPK2 inhibition modulates inflammatory response in both mouse and human macrophages. Remarkably, circHIPK2-silenced iPSCDMs showed therapeutic potential by mitigating inflammation and fibrosis in established myocardial injury. Macrophage-based or macrophage-targeting therapeutic strategies have been extensively investigated in various diseases, extending beyond the heart to include other organs such as the kidney and liver.^58–61^ Our findings suggest that circHIPK2-silenced iPSCDMs hold translational potential as macrophage-based cell therapy. We employed the LMS platform to evaluate circHIPK2 KD macrophage therapy in failing human heart tissue. While LMS enables direct *ex vivo* assessment in human cardiac tissue, its limited culture duration restricts observations to acute or intermediate effects. Additionally, donor variability introduces biological heterogeneity, complicating interpretation of therapeutic outcomes. Although our co-culture model supports testing of cell-based therapies, outcomes may vary with cell attachment efficiency. Thus, careful donor stratification (e.g. age, medication, disease history, fibrotic remodelling) and standardized cell integration protocols are crucial to enhance reproducibility and translational value for cell therapy development.

Specifically, circHIPK2 inhibition reprograms macrophages towards an anti-inflammatory phenotype, promoting cardiac remodelling by facilitating inflammation resolution and fibrosis regulation. Our findings highlight circHIPK2 as a promising therapeutic target for modulating cardiac macrophage and attenuating inflammatory responses associated with MI-induced cardiac injury. To facilitate translation, several key steps are required. First, *in vivo* validation in large animal models, such as pigs, which closely resemble human cardiac anatomy and physiology. Second, comprehensive toxicology and biodistribution studies to evaluate safety, immune tolerance, and durability of therapeutic response. Lastly, optimization of delivery platforms, including AAV capsid engineering, lipid nanoparticles (LNPs), and cardiac macrophage targeted nanocarriers, to achieve efficient and cell-specific delivery with minimal off-target effects.

### Study limitations

This study has several limitations that warrant cautious interpretation. First, the discovery cohort was drawn from end-stage non-ischaemic DCM patients rather than exclusively ischaemic cardiomyopathy (ICM); although an independent ICM dataset confirmed elevated circHIPK2, the absence of detailed aetiology metadata limits the specificity of our conclusions. Second, our analysis is confined to tissue- and cell-specific measurements and lacks longitudinal circulating circHIPK2 data (e.g. during hospitalization, at discharge, and follow-up post-infarction) needed to establish translational and prognostic relevance. While phase-dependent regulation of circHIPK2 is shown in animal models, its role in reperfusion-based clinical scenarios and chronic heart-failure remodelling remains to be explored, raising the need for long-term CRISPR-Cas13-based therapeutic evaluation. Finally, as with any observational design, residual confounding cannot be ruled out; we did not fully account for factors such as age, sex, cardiovascular risk, medication use, or pulmonary comorbidities, which may influence the observed associations.

## Conclusions

In conclusion, this study identifies circHIPK2 as a key regulator of macrophage function and cardiac inflammation, establishing the foundation for innovative therapeutic strategies against MI. While these findings are promising, continued research is needed to fully understand the therapeutic potential of circHIPK2 modulation in cardiovascular disease.

## Supplementary Material

ehaf1116_Supplementary_Data

## Declarations

### Data Availability

All data needed to evaluate the conclusions in the paper are present in the paper and/or the Supplementary Materials. Any materials described in the manuscript can be provided by T.T. pending scientific review and a completed material transfer agreement. Request for the materials should be submitted to the corresponding authors T.T. (Thum.Thomas@mh-hannover.de).
