# Supplementary material for: Macrophage-specific circular RNA circHIPK2, inflammation, and fibrosis after myocardial infarction
Source: Eur Heart J. 2026 Feb 11;47(22):2831–47. doi: 10.1093/eurheartj/ehaf1116 (PMC13247189; doi:10.1093/eurheartj/ehaf1116)
Supplement: ehaf1116_Supplementary_Data [file ehaf1116_supplementary_data.docx]

Supplementary material online

**Table of contents**

| Supplementary Methods | 2 |
| --- | --- |
| Supplementary Figure Legends | 18 |
| Supplementary Figures  Supplementary Tables | 21  26 |
|  |  |
| References | 31 |

**Supplemental Methods**

**Human tissue sampling.**  Ethical approval for the utilization of patient samples in this study was granted by the institutional ethics committee of Hannover Medical School, Germany (project number 9071_BO_K_2020). Cardiac tissue biopsies were obtained from HF patients during either left ventricular assist device (LVAD) implantation or heart transplantation procedures for subsequent use in living myocardial slice culturing (Table S6). Concurrently, plasma was collected from the same cohort of patients for peripheral blood mononuclear cell (PBMC) isolation. This research strictly adhered to the ethical guidelines delineated in the Declaration of Helsinki and its subsequent amendments, as well as comparable ethical standards. All participants were thoroughly informed about the study procedures and voluntarily provided their full written informed consent prior to sample and information collection. The collection and analysis of patient samples, including the procurement of written informed consent, followed established protocols from previous studies^1^.

**RNA-Seq data source (Human tissue_HF vs. Healthy).** The human-tissue RNA-seq dataset employed in this study was derived from a publicly reported cohort ^2,3^, which originally comprised seven explanted left-ventricular samples (6 DCM and 1 TGA). A subset of five samples was used for sequencing^1^. Two samples were excluded after RNA-quality control (Agilent Bioanalyzer RIN < 5.0), leaving five high-integrity samples for RNA-seq analysis. Exclusions were based solely on RNA integrity, independent of clinical parameters. Given the exploratory, hypothesis-generating nature of this study and consistency with other human myocardial RNA-seq investigations, this sample size was considered appropriate. All key RNA-seq findings were subsequently validated by RT-qPCR.

**Animal experiment.** Animal experiments were approved by the authorities at Hannover Medical School and the Niedersächsische Landesamt für Verbraucherschutz und Lebensmittelsicherheit (LAVES) (animal experiment proposals 15/1978, 17/143 and 21/3764). A mouse model of myocardial infarction (MI) was induced in 10-12 weeks old C57BL/6J mice (Charles River Laboratories) through coronary artery ligation, as previously described^4,5^. Briefly, mice were anesthetized with 2% isoflurane, intubated, and artificially ventilated. A 1.5 cm mid-thorax incision was made, the left pectoralis muscle was cut, and thoracotomy was performed between the second and third rib. The left coronary artery was ligated with 8-0 suture, 1 mm distal to the left atrium. Post-surgery, animals received 0.8 mg/mL Metamizole in drinking water for up to 3 days. Successful coronary occlusion was indicated by significant color changes in the ischemic area. The sham procedure was identical, except for the coronary vessel ligation. Specifically, the sample size was estimated through a power calculation (α = 0.05 and 80 % power) based on the expected change in ejection fraction (EF) between treatment groups, derived from our pilot data and previous MI studies. In accordance with the 3Rs principle (Replacement, Reduction, and Refinement), the number of animals was minimized while ensuring adequate statistical power. Accounting for an anticipated ~30% mortality in our post-MI model, we initially enrolled 15 mice per group and followed them for a 28-day endpoint post-MI. Animals that died prior to the endpoint (3 in scr-shR group; 4 in circHIPK2 shR group) or exhibited infarct size below 30% (4 per group) were excluded from further functional and histological analyses. Mice were randomly assigned to experimental groups and monitored according to local regulations. Echocardiographic images were acquired serially and analyzed using standard imaging protocols for global cardiac volumes and function using Vevostrain software (Fujifilm VisualSonics Inc). At the end of experiment, mice were euthanized, and hearts were harvested for further molecular and cellular assays. All experiments adhered strictly to the Animal Research: Reporting of In Vivo Experiments (ARRIVE) guidelines, minimizing animal numbers. Sample size was determined based on empirical data obtained from previous studies and field practices. Mice were assigned unique identifiers and randomly allocated to groups using a standardized protocol.

**Adeno-associated virus (AAV) production, purification, and transduction.**  The AAV vector for circHIPK2 inhibition was designed and packaged by VectorBuilder (VectorBuilder Inc., Chicago, IL, USA). The vector IDs for circHIPK2 and scramble constructs were VB220629-1565kds and VB220913-1125bmc, respectively, which can be used to retrieve detailed vector information from the VectorBuilder database. The mouse circHIPK2 or scramble short hairpin RNA (shRNA) sequences (Table S2) were subcloned into the Mammalian miR30-shRNA Knockdown AAV Vector. Additionally, an enhanced green fluorescent protein (EGFP) tag and CD68 promoter were incorporated into the same vector. HEK 293T cells were transfected with either CD68 promoter-AAV-scramble shRNA or CD68 promoter-AAV-circHIPK2 plasmid, along with helper plasmids (pDP6rs for AAV serotype 6 and pDG9 for AAV serotype 9, kindly provided by Prof. Roger Hajjar, Mount Sinai Hospital, New York) using polyethylenimine (PEI). The next day, the culture medium was replaced with fresh medium, and cells were incubated for 72 hours. Following incubation, the cell pellet was collected in lysis buffer, while the cell culture supernatant was precipitated with 40% polyethylene glycol (PEG) 8000, pH 7.4. The precipitation was carried out at 4°C with constant stirring for 1 hour, followed by overnight incubation at 4°C without stirring. The precipitated virus was collected by centrifugation at 2800 xg for 15 minutes, and the viral pellet was combined with the cell lysate. AAV particles were purified using an iodixanol gradient (OptiPrep, Progen) following benzonase treatment of the lysate. Ultracentrifugation was performed at 63,000 rpm for 1 hour using a Beckman Coulter ultracentrifuge. The 40% iodixanol fraction, containing the purified AAV particles, was further concentrated using Amicon Ultra-15 columns (Millipore). AAV titration was performed using RT-qPCR, targeting the CD68 promoter of the viral genome.

**AAV transduction.** For AAV transduction *in vivo*, mice (8-10 weeks old) were randomly assigned to receive 1x10¹² viral genomes (vg) of AAV9 via intravenous injection. Seven days post-injection, the mice underwent coronary artery ligation. Cardiac function was assessed serially by transthoracic echocardiography using a Vevo 2100 system (Fujifilm VisualSonics Inc.) at baseline (pre-MI), day 7, and day 28 post-MI as described above. The experimental endpoint was set at 28 days post-MI, after which mice were euthanized, and the heart and other organs were collected for further molecular and cellular analyses.

For AAV transduction *in vitro*, Raw264.7 macrophages and mBMDM were transduced by AAV6 at a MOI (Multiplicity of infection) of 2x10^4^ for 72 hours. The transfected cells were then harvested for further experimental use.

**Echocardiography.** Echocardiography was performed on anesthetized mice as previously described^6,7^. Contractile function and heart rate were assessed using the Vevo 3100 system (Fujifilm VisualSonics Inc) before MI and at days 7 and 28 post-MI. Anesthesia was induced with 4% isoflurane (in 100% oxygen), then maintained at 0.5%–1% via a rodent-specific vaporizer and nose cone (Fujifilm VisualSonics Inc). Mice were placed supine on a prewarmed platform, maintaining body temperature at 37°C throughout the procedure. Echocardiographic measurements were obtained 5 minutes post-anesthesia induction when heart rate had stably recovered to exclude the variation in cardiac function created by time after induction. Parasternal short- and long-axis views were recorded in B- and M-mode at the papillary muscle level. Echocardiography image analysis followed previously described methods and adhered to the Guidelines for Measuring Cardiac Physiology in Mice. Left ventricular (LV) end-systolic volume (ESV), end-diastolic volume (EDV), stroke volume (SV = EDV-ESV), ejection fraction (EF = [SV/EDV] x 100), end-diastolic diameter (LVEDD), end-systolic diameter (LVESD), and fractional shortening ([LVEDD–LVESD]/LVEDD x 100) were measured from still images. Cardiac output was calculated using VisualSonics Vevo 3100 software version 3 (Table S4).

**Positron emission tomography molecular imaging of inflammation and fibroblast activation.** To obtain non-invasive measurements of inflammation and fibroblast activation after myocardial infarction after AAV9-circHIPK2-shR therapy, mice underwent sequential positron emission tomography/computed tomography (PET/CT) imaging at 3d using the chemokine CC motif type 2 receptor ligand ^68^Ga-ECL1i and 14d using fibroblast activation protein inhibitor ^68^Ga-MHLL2, respectively^8,9^. Briefly, mice were injected with 11.4±1.3 MBq of ^68^Ga-ECL1i or 12.8±0.8MBq of ^68^Ga-MHLL2 in a bolus of 120µL of saline via a lateral tail vein. After 50 min of conscious uptake, animals were anesthetized with isoflurane (3% at 3L/min oxygen induction, 1.2-1.5% at 0.8 L/min oxygen maintenance) and placed in pairs prone in the scanner bed of a dedicated small animal PET camera (Siemens Inveon DPET). Respiration was monitored continuously and anesthesia concentration adjusted to maintain a constant respiratory rate. A static image was acquired from 50-60min after tracer injection. At the end of the PET acquisition, a short low dose CT scan was acquired for anatomic colocalization. To define myocardial contours and the infarct territory, mice subsequently received intraperitoneal ^18^F-2-fluoro-2-deoxyglucose (FDG, 19.2±2.6 MBq) in 200µL of saline. A second static PET image was acquired from 20-30min after FDG administration.

Images were reconstructed to a 128x128x159 image matrix (0.78 mm pixel size) using an iterative ordered subset expectation maximization, maximum a posteriori algorithm (OSEM3D-MAP) with standard corrections for scatter, attenuation, and radioactivity decay as described previously^10^. PET/CT images were semi-automatically coregistered using Siemens IRW 4.2 software. Regions of interest were manually defined for the infarct and remote myocardium using the ^18^F-FDG viability images and cross-applied to the co-registered ^68^Ga-ECL1i and ^68^Ga-MHLL2 images. The raw voxel intensity (Bq/cm^3^) were used to calculate percent injected dose per gram of tissue (%ID/g) as described previously^10^. Fused images are displayed using AMIDE software.

**Isolation of cardiac macrophages and cardiac fibroblasts.** Mice were euthanized at day 1 and day 7 following MI, with day 0 collected as no-MI controls. LV macrophages were isolated from the infarct region using immunomagnetic separation as previously described^5,11^. Briefly, excised LV tissue was rinsed, minced, and digested with collagenase II (Worthington; Lakewood, NJ) and DNase solution in Hanks buffered saline solution. A single-cell suspension was generated and filtered through a 30-µm pre-separation column. Cell suspensions were incubated at 4°C with anti-Ly6G-biotin antibody for 20 minutes (Miltenyi Biotech, Bergisch Gladbach, Germany, 130-092-332) to remove neutrophils, followed by anti-CD11b-biotin antibody (Miltenyi Biotech, 130-049-601) for 15 minutes, and then anti-biotin microbeads (Miltenyi Biotech, 130-092-332) for 10 minutes. Antibody-microbead conjugated cells were separated using magnetic columns (Miltenyi Biotech, 130-042-201). The isolated macrophages (CD11b^+^Ly6G^-^) were confirmed with cellular markers by FACS analysis and used for transcriptomics analysis by RNA sequencing (RNA-Seq). The effluent (Ly6G- and CD11b^-^) was plated in T25 flasks containing Dulbecco's Modified Eagle Medium (DMEM) supplemented with 10% fetal bovine serum (FBS) and 1x antibiotic–antimycotic solution (Gibco #15240-062). Cardiac fibroblasts at passage 3 were used for scratch assay with secretome collected from macrophages.

**Heart fractionation.** To investigate circRNA expression dynamics within major cardiac cell populations during acute post-myocardial infarction (MI) inflammation, we employed a standardized protocol for cardiac cell isolation and sorting^12^. Adult mice underwent permanent left anterior descending (LAD) coronary artery ligation followed by 24-hour recovery prior to tissue processing. Mice were euthanized at day 1 post-MI. By inserting a blunt needle into the aorta, retrograde perfusion of the heart was performed with perfusion buffer (113 mM NaCl (Sigma), 4.7 mM KCl (Sigma), 600 µM KH2PO4 (Sigma), 600 µM Na2HPO4 Sigma) 1.2 mM MgSO4-7∙H2O (Sigma), 32 µM phenol red (Sigma), 12 mM NaHCO3 (Sigma), 10 mM KHCO3 (Sigma), 10 mM HEPES (Sigma), 10 mM taurine (Sigma), 0.1 % glucose (Sigma) and 10 mM BDM (Sigma), pH 7.46) in vivo as well as ex vivo. Subsequently, hearts were perfused ex vivo with digestion buffer (perfusion buffer supplemented with 12.5 µM CaCl2 (Sigma), 700 U/ml Collagenase type II (Worthington)) until the heart appeared swollen, pale and flaccid. The ventricles were separated from the atria, minced in digestion buffer and sheared before stopping the enzymatic digestion with perfusion buffer supplemented with 10 % FBS (Gibco) and 12.5 µM CaCl2 (Sigma). Cells were sheared once more, filtered through a 100 µm MACS® Smart Strainer (Miltenyi Biotec). The resulting supernatant was transferred into a new tube, while sedimented cardiomyocytes-enriched pellets were snap-frozen in liquid nitrogen. The non-myocyte fractions were isolated by centrifuging the supernatant and transferring it to a new tube. The collected cells were resuspended in cold MACS® buffer (5% MACS® BSA Stock Solution in autoMACS® Rinsing Solution, both from Miltenyi Biotec). The cells were then stained with antibodies targeting specific cell surface markers: cardiac fibroblasts (CD90-APC, eBioscience 17-0900-82), endothelial cells (CD31-APC, eBioscience 11-0311-82) and leukocytes (CD45-PE, eBioscience 12-0451-82). Stained cells were analyzed on a BD FACS Aria III using DAPI exclusion for viability assessment. Post-sorting, CD11b⁺ leukocyte subsets (cardiac macrophages) were further isolated via anti-CD11b-biotin antibody based magnetic separation (Miltenyi Biotech, 130-049-601). All sorted populations underwent snap-frozen in liquid nitrogen and stored in RNA stabilization buffer at -80°C for subsequent molecular analyses.

**Flow Cytometry and Cell Sorting.** To confirm the in vivo targeting efficiency of AAV-mediated delivery, fluorescence-activated cell sorting (FACS) analysis was performed on cardiac cells. Mice were intravenously injected with GFP-tagged AAV9 virus, followed by coronary artery ligation 7 days post-injection. At 7 days post-MI, the mice were euthanized, and hearts were harvested and enzymatically digested to obtain cellular fractions, as previously described (*55*). Briefly, excised cardiac tissue was rinsed, minced, and digested using a collagenase II solution. The homogenized tissue was then passed through a 30-μm pre-separation column to obtain a single-cell suspension. Red blood cells were lysed (Red Blood Cell Lysis Solution, Miltenyi Biotech, 130-094-183), and Fc receptors were blocked using FcR Blocking Reagent (Miltenyi Biotech, 130-092-575) to minimize non-specific interactions. Cells were stained with DAPI for viability assessment and labeled with PE-conjugated CD68 antibody (BD Biosciences) for macrophage identification. FACS analysis identified four distinct subpopulations based on GFP expression and CD68 labeling. Cell sorting was performed using a BD FACS Aria IIu flow cytometer, and data were analyzed using FlowJo software. Proper gating was ensured using no-fluorescence and single-fluorescence controls. Cell populations were identified through a sequential gating strategy and confirmed using specific cellular markers.

**Organ harvest.** Mice were anesthetized with 2% isoflurane in oxygen within an inhalation chamber and euthanized by cervical dislocation. Following disinfection, the thoracic and cranial cavities were opened to harvest the brain, heart, kidneys, liver, lungs, lymph nodes, and spleen. For tissue collection, the heart was flushed with a cardioplegic solution to induce diastolic arrest. The LV was sliced into apex, middle, and base sections, stained with 1% 2,3,5-triphenyltetrazolium chloride (TTC), and imaged for infarct area quantification. Infarct size was calculated using ImageJ and expressed as a percentage of the infarct area relative to the total LV area. The infarct and remote myocardial regions were separately snap-frozen and stored at −80°C for real-time RT-PCR or immunoblotting analysis. The middle LV section was fixed in 10% zinc formalin, paraffin-embedded, and sectioned for histological evaluation.

**Cell culture.** RAW264.7 macrophage cell line was cultured in DMEM supplemented with 10% FBS (Gibco) and maintained at 37°C with 5% CO₂ in a humidified incubator. HEK-293T cells were cultured under similar conditions in DMEM supplemented with 10% FBS and 1% penicillin/streptomycin (P/S), following the manufacturer’s instructions. Mouse bone marrow-derived macrophages (mBMDMs) were harvested from femurs and tibiae of C57BL/6J mice (8-12 weeks old). mBMDMs were cultured in Roswell Park Memorial Institute (RPMI) 1640 medium supplemented with 20 ng/mL recombinant mouse M-CSF (R&D Systems) for 7 days, as previously described^6^. mBMDMs were subsequently rinsed and used for siRNA transfection or overexpression studies of circHIPK2. iPSC-derived macrophages were obtained from AG Lachmann and derived from the previously established hiPSC line ‘Phoenix’^7,13^, following a previously published differentiation protocol^14^. Terminal differentiation into iPSC-derived macrophages was induced by incubating harvested cells for 5 days in RPMI Differentiation Medium [RPMI 1640 (Gibco) supplemented with 10% FBS, 1% L-glutamine (200 mM), 1% P/S (10,000 U/mL), and 50 ng/mL human M-CSF]. Mycoplasma contamination was periodically tested throughout the differentiation process.

**circHIPK2 overexpression plasmid.** The murine circHIPK2 sequence along with its circularization elements was PCR-amplified from HL-1 cell genomic DNA using HotStar Taq Master Mix (Qiagen). EcoRI and XhoI restriction sites were incorporated into the forward and reverse primers, respectively, for further cloning use. The amplified transgene was subsequently subcloned into the pcDNA3.1(+) Laccase2 MCS Exon Vector (Addgene #69893) for further experiments. Primer sequences used in circHIPK2 overexpression plasmid construction can be found in Table S2.

**Plasmid mutation.** The potential binding region between circHIPK2 and G3BP1 was proved by in silico interaction predictions using catRAPID (http://s.tartaglialab.com/page/catrapid_group). To study the functional binding site on circHIPK2 to G3BP1 protein, we introduced three deletions on different circHIPK2 regions, 100-200 bp, 200-400 bp, and 800-900 bp, into the circHIPK2 overexpression plasmid by site-directed mutagenesis using the Q5® Site-Directed Mutagenesis Kit (New England Biolabs, Ipswich, MA, USA, E0554S) following the manufacturer's protocol. The wild-type circHIPK2 overexpression plasmid served as a control group. The primers used for the mutagenesis are listed in Supplementary Table 1. The PCR reaction was performed using an annealing temperature of 61–62 °C for 20 seconds, followed by an extension step at 72 °C for 3 minutes, for a total of 25 cycles. The resulting plasmid was transformed into stabl3 competent cells, and each deletion in the final construct was verified by Sanger sequencing. The resulting mutant plasmids were transfected into RAW264.7 cells to investigate the critical regions of circHIPK2 involved in its interaction with G3BP1.

**Cell transfection and treatment.** For siRNA-mediated knockdown, circHIPK2 and G3BP1 siRNAs were transfected at a final concentration of 100 nM using RNAiMAX (Life Technologies) in OptiMEM medium (Life Technologies) for 24 hours. For overexpression, circHIPK2 plasmids were transfected at a final concentration of 1 μg/mL using Lipofectamine 3000 (Life Technologies) in OptiMEM medium for 48 hours.

For lipopolysaccharide (LPS) stimulation, RAW264.7 macrophages or mBMDMs were treated with 100 nM LPS (Sigma-Aldrich) for 24 hours following transfection. For RNA stability analysis, HL-1 cells and primary neonatal rat cardiomyocytes were treated with Actinomycin D (2 μg/mL, Sigma-Aldrich) for 0.5 to 24 hours to assess transcript degradation dynamics.

For stress granule (SG) induction, RAW264.7 macrophage cells were transfected with the indicated siRNAs or left untransfected as controls. 48 hours post-transfection, cells were treated with 100 µM sodium Arsenite (Sigma-Aldrich) for 1 hour at 37 °C to induce stress granule formation^15^. Following treatment, cells were fixed and subjected to immunofluorescence staining using an anti-G3BP1 antibody to confirm stress granule assembly.

**Living myocardial slice preparation and culture condition.** Myocardial tissue preparation and culture methodologies were implemented as previously described^16-19^. Tissue preparation and slicing were performed using a modified Tyrode's solution containing (in mM): 140 NaCl, 12 KCl, 1 MgCl₂, 0.9 CaCl₂, 10 D-glucose, 10 HEPES, 30 2,3-butanedione monoxime (BDM), and 0.33 NaH₂PO₄·2H₂O (pH 7.4). Left ventricular tissue blocks were sectioned into 300 μm thick slices using a temperature-controlled vibrating microtome (Model 7000SMZ-2, Campden Instruments, Loughborough, UK) maintained at 4°C. Slice culture establishment involved trimming sections to 7×7 mm dimensions followed by adhesion to 3D-printed plastic rings using surgical-grade histoacryl tissue adhesive (B. Braun, Melsungen, Germany; Cat#1050052). Prepared slices were transferred to MyoDish® 1 Tissue Culture Systems (InVitroSys, Gräfelfing, Germany) using established protocols^18^. Culture medium consisted of Medium 199 (Sigma-Aldrich, St. Louis, MO; Cat#M4530) supplemented with 3% penicillin-streptomycin (Gibco, Waltham, MA; Cat#15140) and 1:1000 ITS liquid media supplement (Sigma-Aldrich; Cat#I3146). Physiological conditioning was achieved through simultaneous mechanical and electrical stimulation. Slices underwent cyclic mechanical stretching to 15% of resting length, maintaining sarcomere lengths at 2.1 μm^17,19^. Electrical field stimulation was applied at 0.2 Hz frequency using 3 ms pulses at 20-30 mA amplitude. All experimental procedures were conducted under sterile conditions at 37°C with 5% CO₂ supplementation.

**LMS co-culture with hiPSC-Derived Macrophages**

To assess the therapeutic efficacy of circHIPK2-inhibited macrophages on established failing heart models, we performed co-culture experiments using human induced pluripotent stem cell-derived macrophages (hiPSC-DMs) on living myocardial slices (LMS) prepared from patients with heart failure (Figure 7B). Tissue block was embedded in low gelling temperature agarose (Sigma-Aldrich, #A9414) and cut with precision-cut vibratome (7000smz-2, Campden Instruments) to 300 µm thick sections at a blade advancing speed of 0.02 mm/s, an oscillation frequency of 80 Hz, and an oscillation amplitude of 2 mm as described previously^19,20^. LMS in the size of 7x7 mm were trimmed respecting the longitudinal muscle fiber orientation, glued at both sided to plastic triangles (InVitroSys) and placed into the biomimetic cultivation chambers (BMCC; MyoDish 1 Laboratory Tissue Culture System, InVitroSys) in M199 (Gibco, #11150-059) supplemented with 0.1 % insulin-transferrin-selenium (Sigma-Aldrich, #I3146) and 3 % penicillin-streptomycin (Gibco, #15140-148). LMS were preloaded to 1 mN and electrically stimulated at 50mA/3ms bipolar pulse, 0.2 Hz under continuous agitation of 60 rpm at 37°C, 5% CO2 and cultured for 20-24 hours to allow stabilization of cardiac contractions. Subsequently, the culture medium was replaced, and 1x10^5^ hiPSC-DMs, transfected with either scramble or circHIPK2 siRNA, were seeded onto each LMS. For the time period of seeding the agitation and electrical stimulation of LMS was paused for 5 mins. Prior to seeding, macrophages were resuspended in 10 µL of Medium 199 to enable localized, high-density application. The co-culture was maintained for additional 3-4 days, with medium refreshed and preload readjusted every 48 hours. Throughout the culture period, LMS contractions were continuously monitored and recorded for subsequent analysis. At the end of the experiment, only LMS that maintained regular electrically-induced contractions were collected for downstream analyses, including qPCR and immunofluorescence imaging. This experimental setup allowed for the evaluation of the impact of circHIPK2-inhibited macrophages on cardiac function in the context of heart failure.

**Detection of circHIPK2 localization (RNA-FISH).** Fluorescence in situ hybridization (FISH) for circHIPK2 was performed using the ViewRNA Cell Plus Assay (Thermo Fisher) in Raw264.7 macrophages, following the manufacturer’s instructions. Briefly, Raw264.7 macrophages were fixed and permeabilized using the buffers provided in the kit. Hybridization with circHIPK2-specific probes was carried out for 2 hours at 40°C. Subsequently, sequential incubations with the pre-amplifier mix, amplifier mix, and label probe mix were performed for 1 hour each at 40°C. Imaging was conducted using a Leica confocal microscope (Leica SP8 MP DIVE FALCON) at 63× magnification.

**G3BP1 and circHIPK2 co-localization.** RAW264.7 macrophages were fixed and permeabilized using the buffers provided in the kit. Blocking was performed for 30 minutes with 5% donkey serum in PBS, followed by incubation with a G3BP1 antibody (1:100 dilution, Proteintech) at room temperature (RT) for 2 hours. Subsequently, cells were hybridized with either a circHIPK2-specific probe or a scramble probe for 2 hours at 40°C. Following hybridization, sequential incubations with the pre-amplifier mix, amplifier mix, and label probe mix were carried out for 1 hour each at 40°C. Cells were then incubated with Alexa Fluor 488-conjugated anti-rabbit secondary antibody (1:500 dilution, Invitrogen) and DAPI (1:1000 dilution, Sigma) for 30 minutes. Imaging was conducted using a Leica confocal microscope (Leica SP8 MP DIVE FALCON) at 63× magnification. Z-stack slices indicating co-localization of circHIPK2 and G3BP1 were generated using LAS X software.

**Subcellular fractionation.** A total of 2.5x10⁶ RAW264.7 macrophages were dissociated from the culture dish by trypsinization, washed once with PBS, and pelleted by centrifugation at 500 xg for 5 minutes. The cell pellet was lysed in 200 µL of lysis buffer A [10 mM Tris-HCl, 140 mM NaCl, 1.5 mM MgCl₂, 0.5% NP-40, and 2 mM RNase inhibitor (Qiagen, 1 U/μL)] and incubated on ice for 5 minutes. The lysate was centrifuged at 1000 xg at 4°C, and the supernatant containing the cytoplasmic fraction was transferred to a fresh tube and stored on ice. The remaining cell pellet was washed once with lysis buffer A, resuspended in 200 µL of lysis buffer B [(10 mM Tris-HCl, 140 mM NaCl, 1.5 mM MgCl₂, 0.5% NP-40, 1% Tween-20, 0.5% deoxycholic acid, and 2 mM RNase inhibitor (Qiagen, 1 U/μL)], and incubated on ice for 5 minutes. The lysate was centrifuged at 1000 xg at 4°C, and the pellet was washed once with lysis buffer B. The remaining pellet was collected as the nuclear fraction.

**RNA isolation, PCR & RT-qPCR.** RNA was isolated from cultured cells and heart tissue using Qiazol (Qiagen) according to the manufacturer’s instructions. A total of 500-1000 ng of RNA was reverse-transcribed using random primers provided in the iScript Select cDNA Synthesis Kit (Bio-Rad). PCR amplification was performed using HotStar Taq Master Mix (Qiagen). Quantitative real-time PCR (RT-qPCR) was conducted with iQ SYBR Green Mix (Bio-Rad) on a QuantStudio 7 instrument (Applied Biosystems) using target-specific primer pairs (see Supplemental Table S1). Divergent primers were used for the amplification of circRNAs, whereas linear transcripts were amplified using convergent primers.

**RNA seq (Macrophage_Scr siR vs. circHIPK2 siR).** Total RNA was extracted from RAW264.7 macrophages treated with either scramble siRNA or circHIPK2 siRNA using the miRNeasy Mini Kit (Qiagen). RNA integrity was assessed using an Agilent Bioanalyzer prior to library preparation. A total of 500 ng of RNA per sample was used for mRNA enrichment with the NEBNext® Poly(A) mRNA Magnetic Isolation Module (New England Biolabs), followed by strand-specific cDNA library preparation using the NEBNext® Ultra Directional RNA Library Prep Kit for Illumina (New England Biolabs). Sequencing was performed on an Illumina NextSeq 550 sequencer using a High Output FlowCell for 2x75 bp paired-end reads. Quality-trimmed short reads were aligned to the Mus musculus reference genome (mm10) using STAR (v2.5.0c) with default parameters. FeatureCounts (v1.6.1) was used for gene quantification, with gene annotations obtained from GENCODE (GRCm38.p6, release M17). Normalization and differential expression analysis were conducted using DESeq2 (v2.11.40.2) with default settings^21^.

**Bioinformatics analysis.** Functional analysis of RNA-seq results was conducted using Enrichr online tool for KEGG pathway analysis^22^. Putative upstream modulators of circHIPK2 were predicted using RegRNA2.0^23^. Interaction networks among circHIPK2, Gene Ontology (GO) pathways, and their associated proteins were generated and analyzed using NanoString^24^. Additionally, catRAPID was employed to predict the probability of interaction between circHIPK2 and proteins identified from the NanoString analysis.

**RNA pulldown assay.** A total of 1x10⁶ RAW264.7 macrophages were washed with ice-cold PBS, lysed in 500 μL co-immunoprecipitation (co-IP) buffer, and incubated with 3 μg biotinylated DNA oligo probes (Integrated DNA Technologies) targeting either endogenous or ectopically expressed transcripts (see below table) at RT for 2 hours. Streptavidin C1 magnetic beads (50 μL, Invitrogen) were added to each reaction and further incubated at RT for 2 hours. The beads were then washed gently five times with co-IP buffer to remove non-specific interactions. Proteins bound in the pulldown fraction were analyzed by western blotting, while RNA samples were subjected to RT-qPCR analysis.

|  | pulldown probe sequence (5'→3') |
| --- | --- |
| circHIPK2 | 5′-Biosg-AAACAGATACTACCGGTATGGCCTCAC-3′ |
| Scramble | 5′-Biosg-AAAAGGTAGTGTAATCGCCTTG-3′ |

**Mass spectrometry analysis.** Protein sample preparation and mass spectrometry experiments were conducted as previously described^25^. RNA pulldown samples were alkylated with 4% acrylamide (Applichem) and separated on a 4-15% Mini-PROTEAN® TGX™ Precast Protein Gel (Bio-Rad). Proteins were stained using Coomassie Brilliant Blue G-250 (Thermo Fisher) and destained by washing twice with double-distilled water (ddH₂O). Gel lanes were excised into four sections, and in-gel trypsin digestion was performed. The resulting digested peptides were extracted and analyzed using an Orbitrap mass spectrometer (MS) (Thermo Fisher). Raw data processing was performed using MaxQuant^26^ and Perseus^27^ software. Protein identification was conducted using the UniProt reviewed mouse proteome database, with a false discovery rate (FDR) threshold of ≤0.01 applied at both the protein and peptide levels. Protein intensities were median-normalized to correct for sample loading variations, and only proteins identified in all biological replicates were considered for quantification. Enriched proteins were identified by normalization to both the negative control (NC) probe and input samples to determine potential binding partners (Supplementary Table S2).

**Immunoprecipitation (IP).** RAW264.7 macrophage cell lysates were subjected to immunoprecipitation using 1 µg of mouse control IgG antibody (Cell Signaling Technology) or 1 µg of mouse anti-G3BP1 antibody (Proteintech). Antibodies were conjugated to Dynabeads™ Protein G (Invitrogen) according to the manufacturer’s protocol. For each IP reaction, lysates from 1 × 10⁷ cells were incubated with antibody-conjugated beads overnight at 4 °C with gentle rotation. Following incubation, beads were washed five times with co-IP buffer to reduce nonspecific binding. The immunoprecipitated material was then processed for downstream analyses: protein fractions were analyzed by western blotting, and co-precipitated RNA was isolated and analyzed by RT-qPCR.

**Western Blotting.** Cell pellets were lysed in 1x Cell Lysis Buffer (Cell Signaling), and total protein concentration was quantified using Roti-Quant (Roth). For each sample, 15-30 μg of protein was loaded onto an SDS-polyacrylamide gel for electrophoretic separation. Proteins were subsequently transferred onto a polyvinylidene fluoride (PVDF) membrane using the Mini PROTEAN Tetra Cell system (Bio-Rad). Specific protein detection was performed using the following primary antibodies: anti-G3BP1 (1:5000, Proteintech), DDX3X (1:1000, Abcam), Caprin1 (1:5000, Proteintech), HNRNPK (1:1000, Proteintech), FUS (1:5000, Proteintech), VINCULIN (1:2500, Sigma). HRP-conjugated secondary antibodies (Cell Signaling) were used for chemiluminescent detection. Band intensity was quantified using Image Lab software (Bio-Rad).

**TUNEL staining.** Cryosections of the heart were fixed in 4% paraformaldehyde for 20 minutes at RT, followed by permeabilization with ice-cold 0.1% Triton X-100 in PBS for 2 minutes at RT. Subsequently, the sections were incubated with the enzyme labeling solution provided in the In Situ Cell Death Detection Kit (Roche) for 1 hour at 37°C. For negative controls, the enzyme was omitted from the labeling solution. After two times PBS washes, the sections were counterstained with DAPI (1:1000 dilution, Sigma) for 15 minutes. Imaging was performed using a Nikon Eclipse Ti microscope, and image analysis was conducted with NIS Elements software. For each heart, 5-10 images were randomly captured from different regions of the section. TUNEL-positive nuclei were counted in each field, and the average number of TUNEL-positive cells per heart was quantified and plotted.

**Scratch assay.** Cardiac fibroblasts isolated 7 days post-MI were seeded directly into Ibidi µ-Slides at a density of 10,000 cells/cm² (70,000 cells/mL) per well. Cells were incubated overnight in DMEM-F12 containing 10% FBS to allow attachment. After adhesion, Ibidi inserts were removed to create an open wound area, followed by PBS washing and incubation with conditioned medium collected from mBMDMs transfected with either circHIPK2 siRNA or scramble siRNA. Live-cell imaging was performed every 6 hours for a total duration of 24 hours using a Cytation 5 Cell Imaging Reader. Wound closure was assessed via high-throughput image analysis using the ImageJ plugin^28^. The cell migration rate and percentage of wound closure were calculated as previously described^28^. Briefly, the difference between the initial wound area and the wound area after n hours was normalized to the initial wound area, measured in µm².

**Cytokine profiling.** Conditioned medium was collected from RAW264.7 macrophages transfected with either circHIPK2 siRNA or scramble siRNA. Supernatant samples were pre-processed by centrifugation at 800 xg for 7 minutes at 4°C. Secreted protein levels were quantified using the Bio-Plex Pro™ Rat Cytokine 23-Plex Assay (Bio-Rad, Hercules, USA, 10000092045), following the manufacturer’s instructions. The assay was performed using the Bio-Plex 200 system, and data were analyzed using Bio-Plex Manager™ version 6.2 software (Bio-Rad, Hercules, USA). Measurements below the detection limit were set to the lowest standard value, while values exceeding the detection limit were set to the highest standard concentration. Cytokines with levels below the detection threshold in more than 50% of samples were excluded from statistical analysis. A heatmap of the analyzed data was generated using MetaboAnalyst 5.0^29^.

**NF-κB Luciferase Reporter Assay**

To assess inflammatory NF-κB signaling activity, a luciferase reporter assay was performed in RAW264.7 macrophages. A stable circHIPK2 knockdown RAW264.7 cell line (circHIPK2 KD) was generated via lentiviral (LV) transduction, and LV-scramble-transduced cells were used as controls (Ctrl). Cells were seeded in 24-well plates at a density of 1 × 10^5^ cells per well. For NF-κB transcriptional activity detection, cells were co-transfected with the pSGNluc reporter plasmid—containing multiple NF-κB binding sites—and a β-galactosidase control plasmid (Promega, USA). 48 hours post-transfection, the medium was replaced with serum-free medium, with or without lipopolysaccharide (LPS; 100 ng/mL). Cells were incubated for an additional 6 hours at 37 °C and 5% CO_2_. Luminescence was measured using a Synergy HT Multi-Detection Microplate Reader (BioTek, USA). Luciferase activity was normalized to β-galactosidase activity to control for transfection efficiency.

**Histology and immunostaining. Tissue)** For histological assessment of fibrosis, paraffin-embedded LV sections were stained with picrosirius red, and collagen content was quantified as the percentage of fibrotic area per LV section. For inflammation analysis, LV sections were stained with hematoxylin and eosin (H&E). For further immunohistochemistry (IHC) or immunofluorescence (IF) staining of tissue, formalin-fixed, paraffin-embedded (FFPE) heart sections (3 µm thick) underwent antigen retrieval using Target Retrieval Solution (S1699, Dako). For IHC staining of infiltrated immune cell population, the sections were incubated with 3% H2O2 (Sigma) to block endogenous peroxidase activity, followed by blocking with goat serum blocking solution (Vector Laboratories). The primary antibody specific for immune cells, CD45 (1:100, Abcam) was used, followed by incubation with goat anti-rabbit IgG and ABC reagent (Vector Laboratories). The DAB substrate kit (Vector Laboratories) was used for positive staining of CD45, and hematoxylin was used as counterstain. The primary antibodies used for IF staining included anti-G3BP1 (1:100, Proteintech), Mac3 (1:100, MyBioSource), CD86 (1:50, Abcam), CD163 (1:50, Abcam). A fluorophore-conjugated secondary antibody against the primary antibody was applied. **Cells**) RAW264.7 macrophages were fixed in 4% paraformaldehyde for 10 minutes and permeabilized with 0.1% Triton X-100 for 10 minutes at RT. Blocking was performed for 30 minutes using 5% donkey serum in PBS, followed by overnight incubation at 4°C with a G3BP1 antibody (1:1000, Proteintech). The next day, cells were washed three times with PBS and incubated with Alexa Fluor 488-conjugated anti-rabbit secondary antibody (1:500, Invitrogen) and DAPI (1:1000, Sigma) for 30 minutes. Images were captured using BZ-X810 inverted fluorescence microscope (Keyence) or Leica SP8 inverted confocal microscope and analyzed with FIJI ImageJ (version 1).

**Statistics.** Data are presented as mean ± SD or as mean with 95% CI where indicated. Comparisons between two groups were assessed using unpaired two-tailed Student’s t-tests. For comparisons among three or more groups, one-way ANOVA followed by Tukey’s post-hoc tests was applied. When evaluating interactions between multiple factors, two-way ANOVA with Tukey’s multiple-comparisons correction was used as appropriate. Summary measures of kinetic data were quantified using area-under-the-curve (AUC) analysis. Correlation analyses were performed using Spearman’s rank correlation. Survival analyses were conducted using Kaplan–Meier curves with log-rank testing. Normality and homogeneity of variance were assessed for all comparisons. A p-value < 0.05 was considered statistically significant. Exact p-values (reported to 1–2 significant digits) are provided in the figures, whereas p > 0.1 are not reported.

**
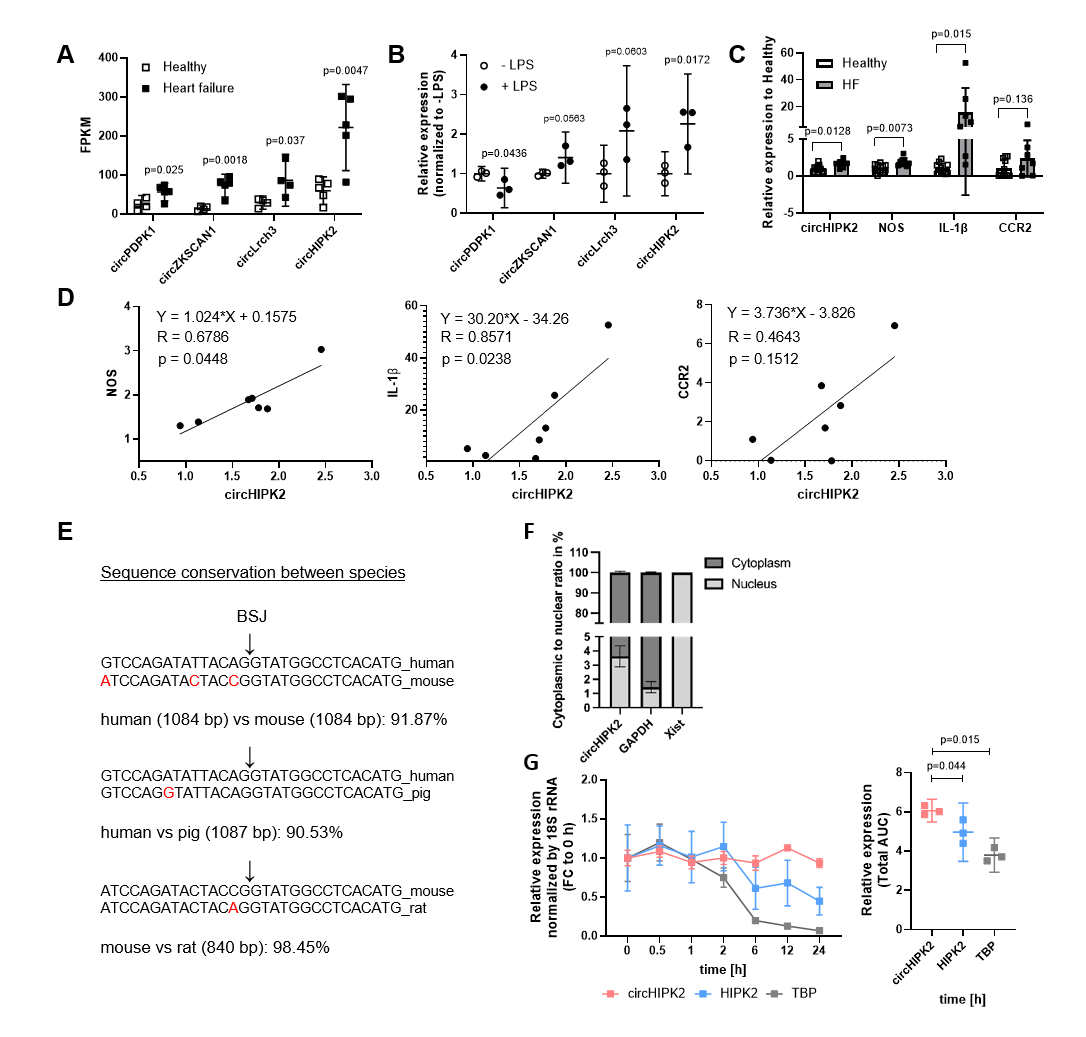
**

**Supplementary Figure 1. circHIPK2 is conserved across species and is highly stable.** (A) Relative FPKM expression of circRNAs (circPDK1, circZKSCAN1, circLrch3, circHIPK2) in failing heart compared to healthy heart tissue. Data are shown as means ± 95% CI (n=3 per group). Statistical comparisons between HF and healthy controls were performed by multiple unpaired t-tests.  FPKM Fragments Per Kilobase per Million mapped fragments. (B) Relative expression of circRNAs (circPDK1, circZKSCAN1, circLrch3, circHIPK2) in Raw264.7 macrophages upon LPS stimulation compared to non-activated macrophages. Indicated p-values were calculated by multiple unpaired two-tailed t-tests comparing conditions with and without LPS. (C) Relative expression of circHIPK2, NOS, IL-1b and CCR2 in failing heart tissue (n=7) compared to healthy controls (n=11). Data are shown as means ± SD. p values were calculated using unpaired t-tests. (D) Correlation between normalized expression between circHIPK2 and inflammatory genes (NOS, IL-1b and CCR2) in failing hearts. Correlation analysis has been performed by Spearman correlation. (E) Sequence alignment of the human, mouse circHIPK2 full sequences (human, mouse: 1084 bp, pig: 1087 bp, rat: 840bp) indicates a high degree of conservation (human vs. mouse 91.87%, human vs. pig 90.53%, mouse vs. rat 98.45%). (F) Subcellular circHIPK2 expression in Raw264.7 macrophages after normalization to the respective subcellular fraction (GAPDH applied for normalization of cytoplasmic fraction, Xist applied for normalization of nuclear fraction, n=3 per group duplicates of three independent experiments). (G) Relative expression of circHIPK2, HIPK2, and TBP in Raw264.7 macrophages treated with Actinomycin D (n = 3 per timepoint). Time-course of circHIPK2 gene expression over 24 h post-treatment (left). Quantification of the overall response using AUC as a summary measure (right). Data are presented as mean ± 95% CI. Statistical analysis was performed using one-way ANOVA with Tukey’s multiple-comparisons test. Exact p-values are shown for the indicated comparisons.

**
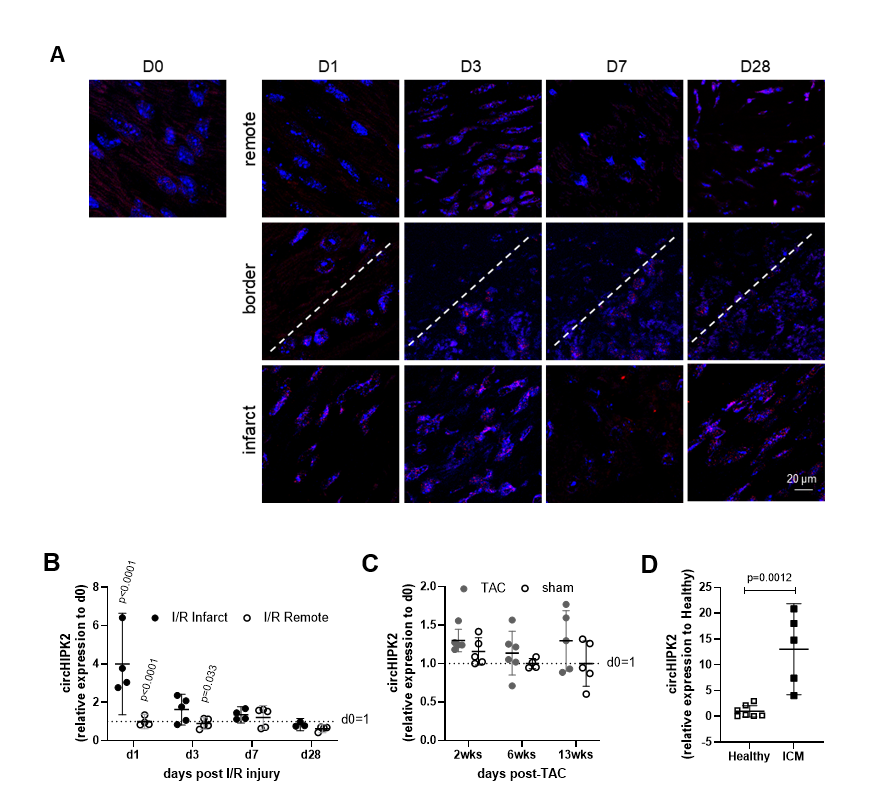
**

**Supplementary Figure 2. circHIPK2 expression in MI, mouse I/R model and human ICM.** (A) RNA-FISH analysis of circHIPK2 expression in mouse heart tissue at d0 (sham), day 1, 3, 7, and 28 following MI, using ViewRNA probes. circHIPK2 signals (red) are prominently detected within 1 week of post-MI at infarct region. Nuclei were stained with DAPI (blue). Representative images were acquired from three independent tissue samples per group, with five fields captured per section. (B) RT-qPCR quantification of circHIPK2 in myocardial tissue from a mouse ischemia/reperfusion (I/R) model. Closed circles indicate the infarct region and open circles indicate remote myocardium (n=3-5). Expression values are normalized to day 0 (dotted line = 1). Statistical comparisons: p < 0.001 (d0 vs. d1), p < 0.001 (d1 infarct vs. remote), and p = 0.033 (d3 infarct vs. remote). Data are shown as mean ± 95% CI. (C) RT-qPCR quantification of circHIPK2 in a transverse aortic constriction (TAC) model (closed circles) versus sham (open circles), normalized to day 0 (dotted line = 1). Data are shown as mean ± 95% CI (n=4-6). (D) Tissue expression of circHIPK2 in human myocardium quantified by RT-qPCR (healthy n=7; ICM n=5). Comparisons between healthy and ischemic oriented tissue (ICM) samples were performed using an unpaired two-tailed t-test. Exact p-values are indicated.

**
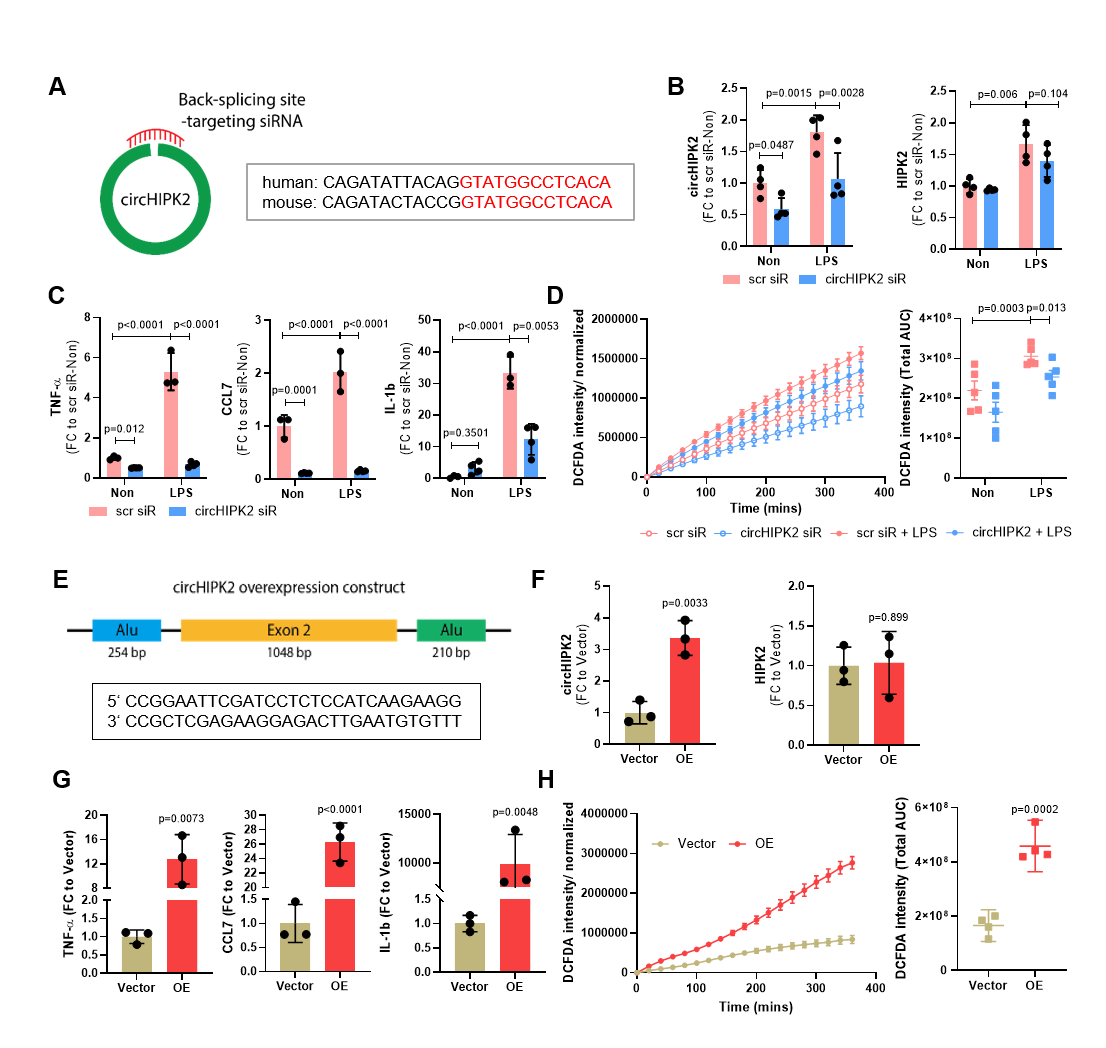
**

**Supplementary Figure 3. Loss and gain of circHIPK2 function on inflammatory gene expression and ROS production in macrophages.** (A) CircHIPK2 siRNAs were designed spanning the BSJ (left). Relative sequence of siRNA for human and mouse were aligned (right). (B) Relative expression of circHIPK2 and HIPK2 in mBMDM transfected with circHIPK2 siRNA compared to scr siRNA with and without LPS induction (n=4). (C) Relative expression of inflammatory genes (TNF-α, CCL7, and IL-1b) in mBMDM transfected with circHIPK2 siRNA compared to scr siRNA with and without LPS induction (n=4). (D) Time course of DCFDA in Raw264.7 macrophages upon transfection with either scr siRNA or circHIPK2 siRNA, with and without addition of LPS stimulation. Cellular ROS production was monitored every 20 mins for a period of 6 h at 37°C (left). The overall ROS response was quantified as the AUC (right) (n=5). (E) Schematic representation of the overexpression cassette sub-cloned into the pcDNA plasmid. The overexpression cassette consists of the endogenous circHIPK2 exon and circularization elements (ALU) elements cloned from genomic DNA of Raw264.7 macrophages (upper). The primer sequence of overexpression insert is aligned (bottom). (F) Relative expression of circHIPK2 and HIPK2 in mBMDM transfected with the circHIPK2 overexpression plasmid (OE, pcDNA-circHIPK2) compared to empty vector (Vector, pcDNA-empty) (n=5). (G) Altered expression of inflammatory genes (TNF-α, Cxcl10, and IL1RN) in mBMDM following overexpression of circHIPK2 (n=3). (H) The effect of overexpression of circHIPK2 on ROS production were examined by cellular DCFDA intensity. Cellular ROS production was monitored every 20 mins for a period of 6 h at 37°C. The overall ROS response was quantified as the AUC (right) (n=3). BSJ: back-splice junction; mBMDM: mouse bone marrow-derived macrophages. Data are displayed as mean ± SD (B, C, F and G), and mean ± 95 % CI (D and H). p-values were determined using two-way ANOVA with Tukey’s post-hoc test (B–D), and unpaired two-tailed t-tests (F–H). Exact p-values are shown for the indicated comparisons.


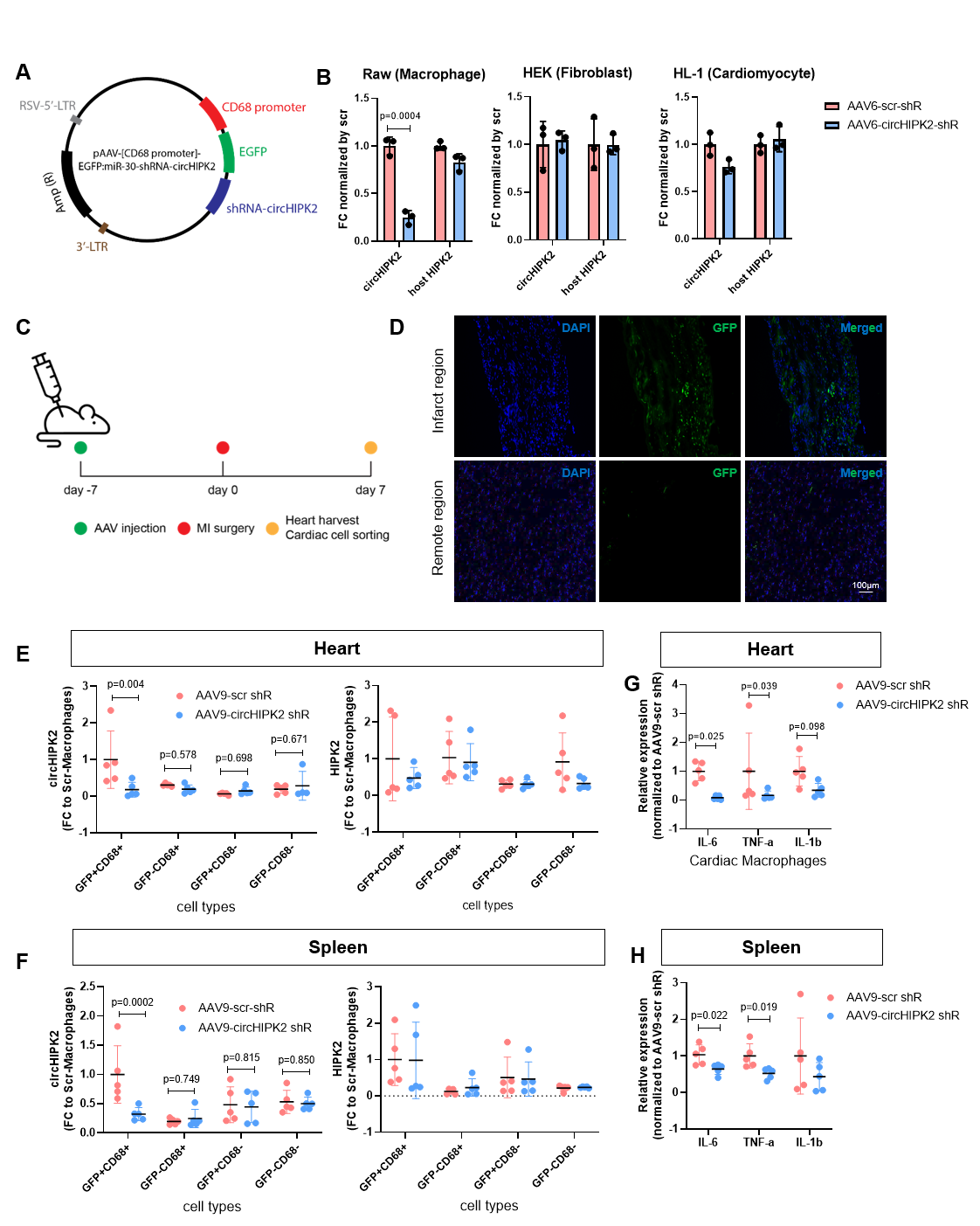


**Supplementary Figure 4. Design of macrophage-targeting AAV9 virus and macrophage-specific reduction of circHIPK2.** (A) Schematic representation of the AAV vector consists of circHIPK2 shRNA or scramble shRNA, together with CD68 promoter and EGFP tag. (B) Relative expression of circHIPK2 following AAV6 transduction in various cardiac cell types, including Raw264.7 macrophages, HEK fibroblasts, and HL-1 cardiomyocytes (n=3). (C) Schematic representation of the *in vivo* animal model to study delivery effects of the AAV9 virus. (D) Visualization of AAV9 vector distribution in post-MI myocardium. AAV9 vectors (1 × 10^12^ vg per mouse) expressing EGFP were administered to the myocardium. EGFP expression (green) was assessed in both the infarct and remote regions 14 days post-injection and 7 days post-MI. Nuclei were counterstained with DAPI (blue). Scale bar = 100 μm. Images are representative of 4-5 fields of view from n = 4 biological replicates. (E-F) Relative expression of circHIPK2 has been measured via RT-qPCR in isolated cells (GFP^+^CD68^+^ macrophages; GFP^-^CD68^+^, GFP^+^CD68^-^, GFP^-^CD68^-^) from both heart and spleen respectively (n=7-8 per group). (F-G) Relative expression of the inflammatory genes (IL-6, IL-1β, and TNF-α) in the isolated macrophages from both heart and spleen comparing AAV9-circHIPK2 shR group with AAV9-scr shR group (n=5 per group). Data are shown as mean ± SD. Statistical analyses were performed using two-way ANOVA with Tukey’s multiple-comparisons test (B, E–F) and multiple unpaired two-tailed t-tests for individual gene comparisons (G–H). Exact p-values for each indicated comparison are shown.

**
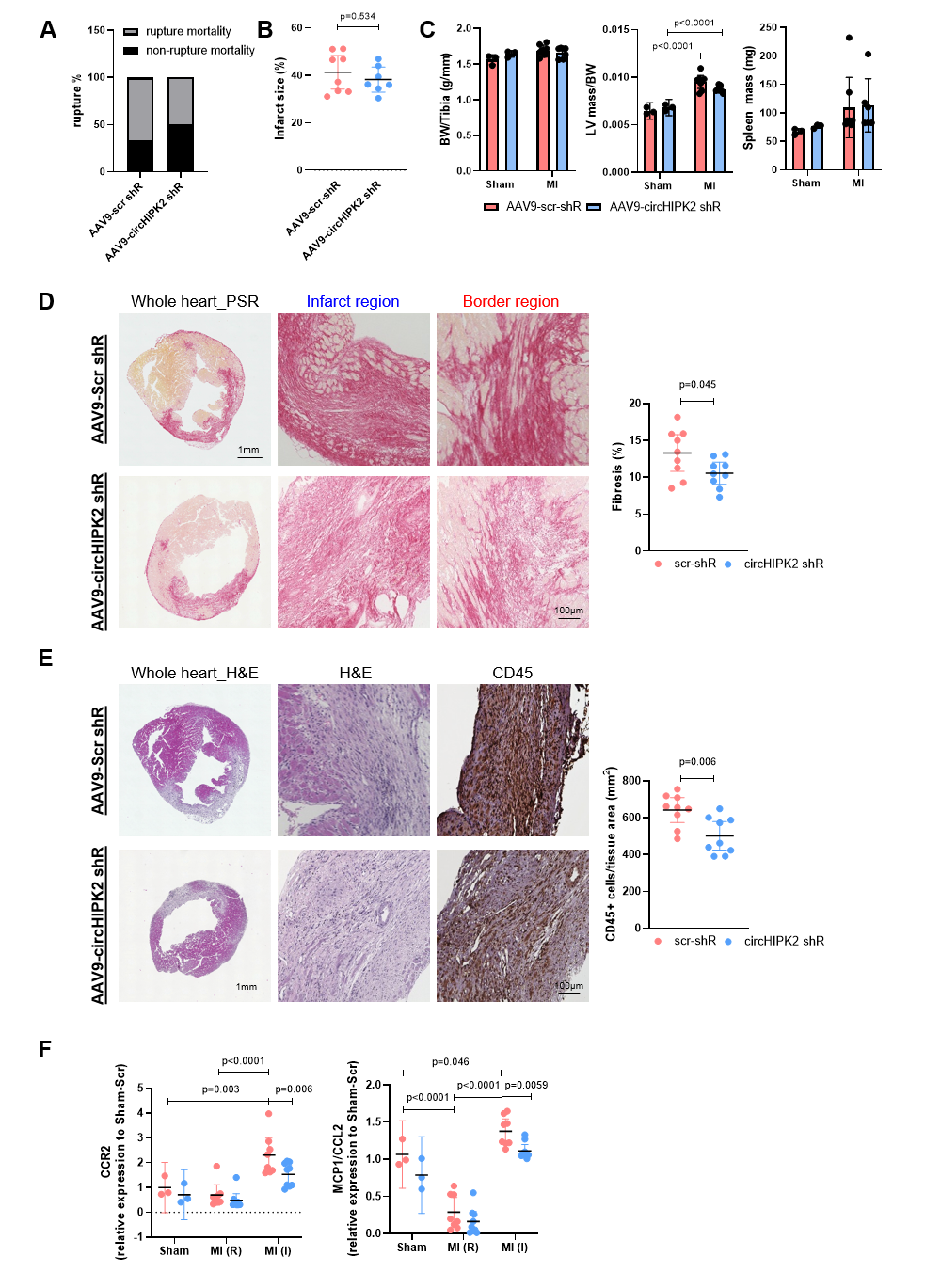
**

**Supplementary Figure 5. The effects of AAV9-circHIPK2 shRNA on early post-MI remodeling.** (A) Rupture rate of animal was displayed as % of the ratio between rupture mortality and non-rupture mortality. In the scramble (scr) group, 2 of 3 mice (66.7%) experienced cardiac rupture, while in the circHIPK2 group, 2 of 4 mice (50%) ruptured. For the remaining censored animals (non-rupture deaths), no specific cause of death could be determined. (B) Infarct size was calculated as the ratio between whole heart and infarcted region in % from TTC heart image (n=7-8 per group). (C) Body weight, LV mass, and spleen mass were measured at the end point (day 28 post-MI) of animal study (n=7-8 per group). (D) Picrosirius red staining (PSR) for fibrosis assessment in heart tissue across four experimental groups (AAV9-scr-shR sham, AAV9-circHIPK2-shR sham, AAV9-scr-shR MI, AAV9-circHIPK2-shR MI) at 7 days post-MI (n=9 per group). Whole heart images (4x magnification) showing left ventricular fibrosis (dark pink) (left upper panel). Scale bar = 1mm. Higher magnification (20x) of infarct and border regions (left lower panel). Scale bar = 100μm. Quantification of collagen deposition per whole heart (right, bar graph). (E) Representative images of H&E-stained heart sections (left panel: whole heart at 4× magnification; middle panel: corresponding higher magnification at 20×) and CD45 immunohistochemistry (right panel) from each experimental group (n=9 per group; scale bar = 100 µm). The right bar graph presents quantitation of CD45⁺ inflammatory cell infiltration per tissue area (mm²). (F) RT-qPCR analysis of CCR2 and CCL2 gene expression in myocardial tissue at 28 days post-MI, comparing infarct and remote regions of post-MI hearts with sham controls. Data are presented as mean ± 95% CI (B, D, and F) or mean ± SD (C). Statistical analyses were performed using unpaired two-tailed t-tests (B, D–E) or two-way ANOVA with Tukey’s multiple-comparisons test. Exact p-values for the indicated comparisons are shown.


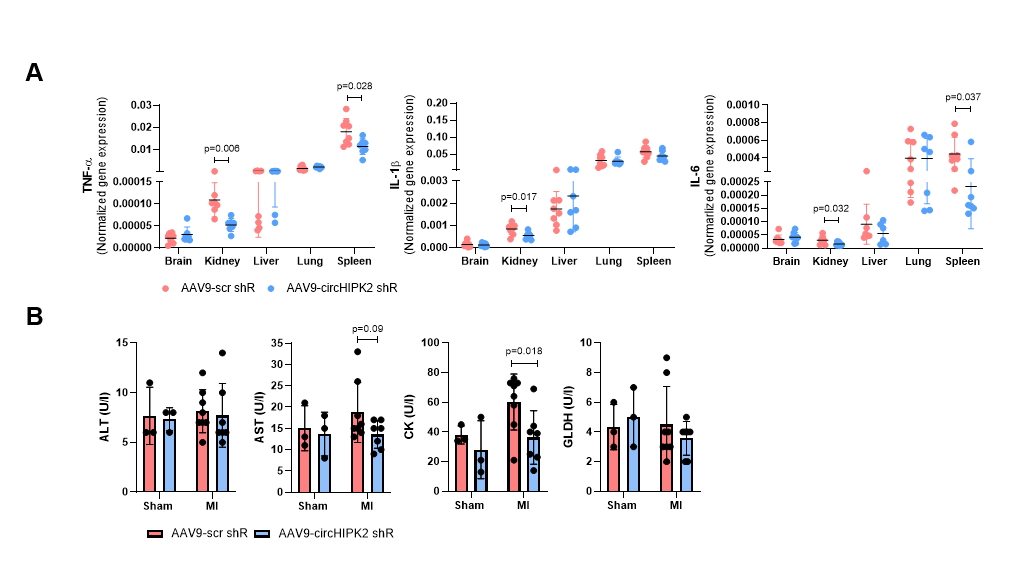


**Supplementary Figure 6. The effects of AAV9-circHIPK2 shRNA on plasma biochemistry and inflammatory response in other organs.**

(A) Relative inflammatory gene expressions (TNF-α, IL-1β, and IL-6) were evaluated in other organs (Brain, Kidney, Liver, Lung and Spleen) at the end point (day 28 post-MI) of the animal study (n=7-8 per group). (B) Plasma level of ALT, AST, CK, and GLDH were evaluated by plasma biochemistry (n=7-8 per group). TTC: triphenyl tetrazolium chloride; ALT: alanine aminotransferases; AST: aspartate aminotransferases; CK: creatine kinase; GLDH: glutamate dehydrogenase. Data are presented as mean ± 95% CI. Statistical significance was assessed using two-way ANOVA with Tukey’s multiple-comparisons test. Exact p-values for indicated comparisons are shown.


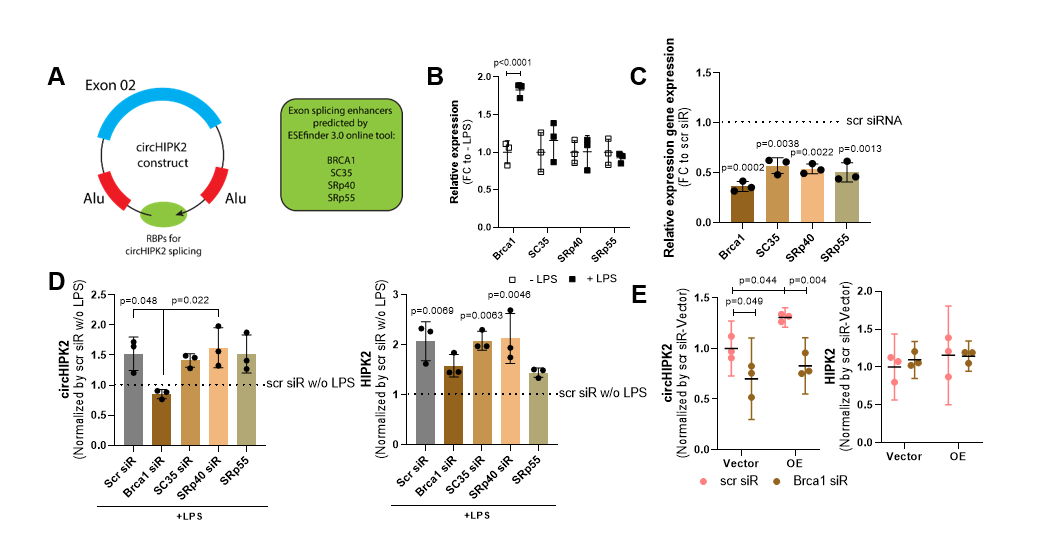


**Supplementary Figure 7.** **Brca1 is an upstream regulator of circHIPK2**

(A) Predicted upstream regulators of circHIPK2 identified using ESEfinder 3.0. (B) Relative expression of Brca1, SC35, SRp40, SRp55 in Raw264.7 macrophages with or without LPS stimulation (n=3). (C) Relative mRNA expression of the indicated transcription factors in RAW264.7 macrophages transfected with siRNAs targeting Brca1, SC35, SRp40, or SRp55 (n=3). The dashed line represents the normalized expression level of 1.0. (D) Relative expression of circHIPK2 and linear HIPK2 transcripts in RAW264.7 macrophages transfected with the indicated siRNAs, with or without LPS stimulation (n = 3). The dashed line represents the normalized expression level of 1.0. (E) Relative expression of circHIPK2 and HIPK2 in macrophages co-transfected with a circHIPK2 overexpression plasmid and either Brca1 siRNA or scramble siRNA. An empty pcDNA vector was used as the control. All data are presented as mean ± SD (B–D) or mean ± 95% CI (E). Statistical significance was assessed by two-way ANOVA with Tukey’s multiple comparisons test (B and E), and by one-way ANOVA with Tukey’s multiple comparisons (G–H). Exact p-values are reported.


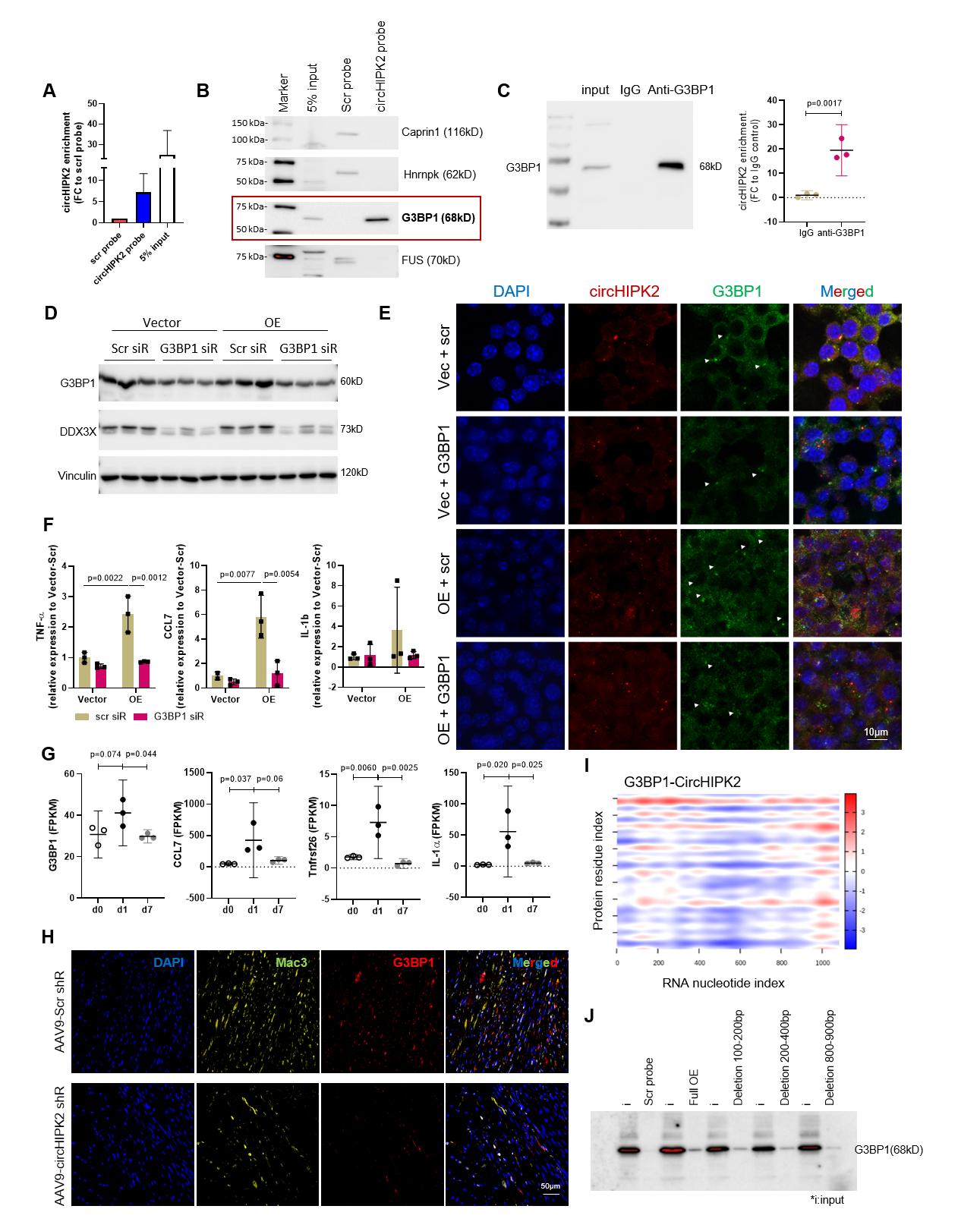


**Supplementary Figure 8. Validation of the interaction between circHIPK2 and G3BP1.** (A) Relative expression of circHIPK2 in Raw264.7 macrophages after circHIPK2 and scramble RNA pulldown using biotin labelled DNA probes (n=3 independent experiments). (B) Western blot analysis of Caprin1, Hnrnpk, G3BP1, and FUS protein levels in RAW264.7 macrophages following RNA pulldown using biotin-labeled DNA probes targeting circHIPK2 or a scrambled control sequence. The assay was performed to assess direct binding of these candidate proteins to circHIPK2. (C) RNA immunoprecipitation was performed in RAW264.7 macrophage cells using either an anti-G3BP1 antibody or IgG isotype control. Western blot (left) shows G3BP1 enrichment in the immunoprecipitated fraction compared to input and IgG control. The bar graph (right) displays RT-qPCR quantification of circHIPK2 in the RIP samples, demonstrating specific enrichment in the G3BP1-IP fraction relative to the IgG control (n = 3 independent experiments). (D) Western blot analysis showing the effects of circHIPK2 overexpression, with or without co-expression of G3BP1, on the protein levels of G3BP1, the stress granule-associated protein DDX3X, and Vinculin as a loading control. (E) Representative RNA-FISH image showing circHIPK2 localization (red) in RAW264.7 macrophages, co-stained with G3BP1 immunofluorescence (green). White arrowheads indicate cytoplasmic stress granules. (Scale bar: 10 μm). Nuclei were stained with DAPI (blue). (F) Relative inflammatory gene expression (TNF-α, CCL7, and IL-1b) was analyzed in mBMDM transfected with circHIPK2 OE plasmids and G3BP1 siRNA (n=3). (G) Relative expression (FPKM) of stress granule related genes (G3BP1, DDX3X) as well as subsequent inflammatory genes (TNF-α, CCL7, IL-1a) were quantified using RNA-seq analysis of the isolated cardiac macrophages at different time points following MI (d0: no infarct; d1 and d7 post-MI) (n=3). (H) Representative immunofluorescence micrographs demonstrating co-localization of infiltrating macrophages (Mac-3) and stress granule (G3BP1) within infarct region of post-MI myocardium Scale bar = 50 μm. Images are representative of 6-10 fields of view from n = 7-8 biological replicates per group. (I) Heatmap of the potential binding regions between circHIPK2 and G3BP1 via CatRAPID. (J) Different regions of circHIPK2, 100-200 bp (lane 6), 200-400 bp (lane 8), and 800-900 bp (lane 10), were mutated using NEB site-directed mutation kit. Afterwards, control (lane 2), WT circHIPK2 (lane 4), and all three mutated circHIPK2 were subjected to RNA pulldown followed by western blot. The lanes labelled with “i” are input control. Data are presented as mean ± 95% CI (C, G) or mean ± SD (F). Statistical analyses were performed using an unpaired two-tailed t-test (C), two-way ANOVA with Tukey’s multiple-comparison test (F), and one-way ANOVA with Tukey’s multiple-comparison test (G) for the indicated comparisons. Exact p-values are shown.


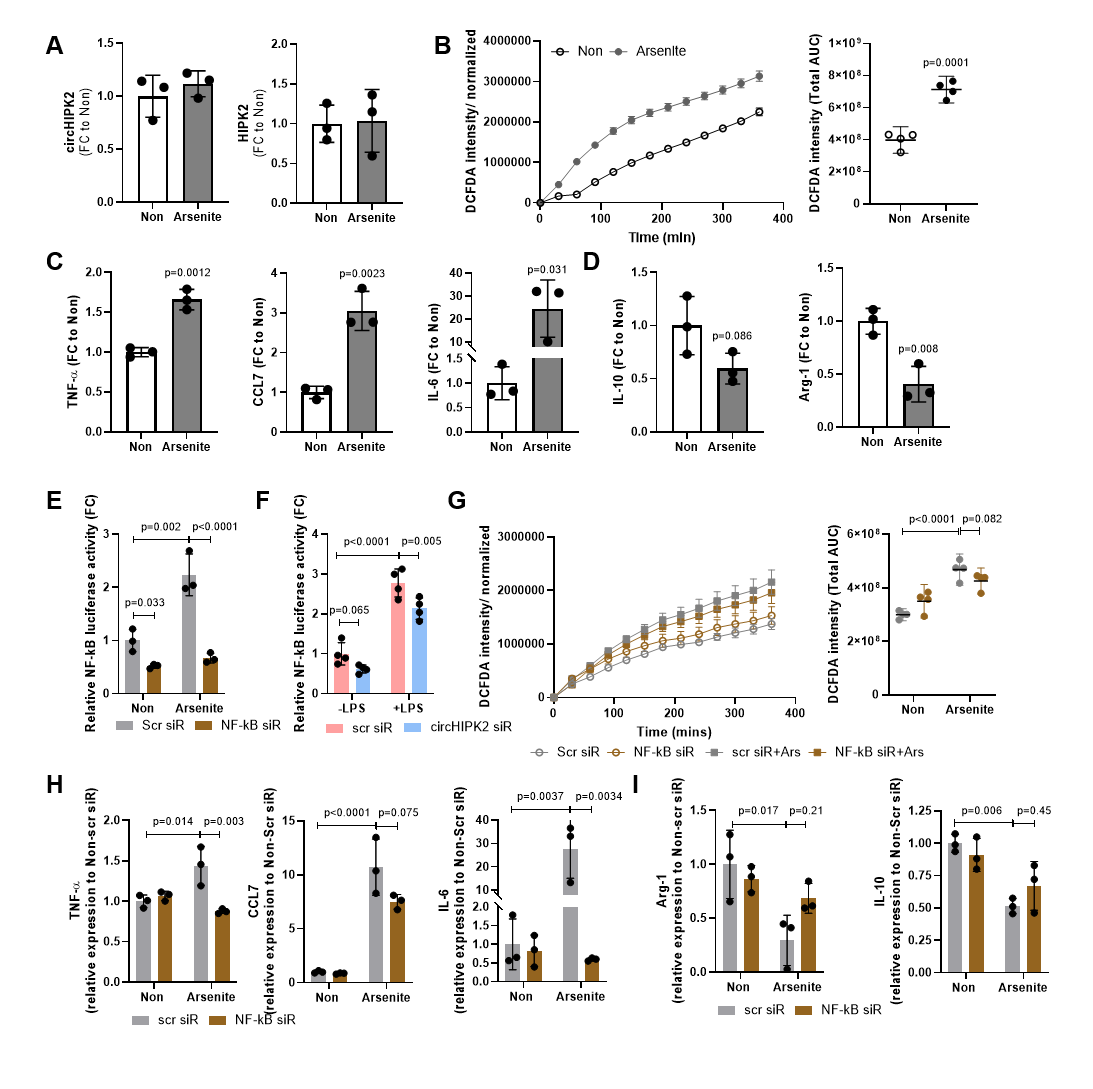


**Supplementary Figure 9. Stress granule formation enhances pro-inflammatory responses in macrophages, with NF-κB acting as a key mediator linking SG formation to inflammatory cascade.** (A) Arsenite stimulation did not alter the expression of circHIPK2 (left) or its host gene HIPK2 (right) in RAW264.7 macrophages. Data are presented as mean ± SEM (n=3). (B) Time-course analysis of ROS production in RAW264.7 cells following Arsenite treatment, measured using DCFDA fluorescence every 20 minutes for 6 hours at 37 °C (left; n=4). The summary ROS response was quantified as the area under the curve (AUC) (right). (C-D) Relative expression of pro-inflammatory genes (TNF-α, CCL7, IL-1β) (C) and anti-inflammatory genes (Arg-1, IL-10) (D) in RAW264.7 cells with or without Arsenite treatment (n=3). (E) Luciferase reporter assay showing NF-κB activity after siRNA-mediated knockdown of NF-κB in the presence or absence of Arsenite treatment (n=3). (F) Luciferase reporter assay of NF-κB activity following circHIPK2 knockdown with or without LPS stimulation (n=4). (G) ROS production over time in cells transfected with scrambled or NF-κB siRNA, with or without Arsenite treatment. ROS levels were measured using DCFDA every 20 minutes for 6 hours at 37 °C (left; n=3). The summary ROS response was quantified as the area under the curve (AUC) (right). (H–I) Relative expression of pro-inflammatory genes (TNF-α, CCL7, IL-1β) (H) and anti-inflammatory genes (Arg-1, IL-10) (I) following transfection with scrambled or NF-κB siRNA, in the presence or absence of Arsenite (n=3). Data are presented as mean ± SD (A, C–F, H) and mean ± 95% CI (B, G). Statistical significance was determined using unpaired two-tailed t-tests (A–D) or two-way ANOVA with Tukey’s multiple-comparison correction (E–I). Exact p-values for the indicated comparisons are reported.

**Supplementary Table 1: Primer list.**

| ***Primer name*** | ***Forward (5'→3')*** | ***Reverse (5'→3')*** |
| --- | --- | --- |
| mmu circHIPK2 | ATATTCGCCCAGTCCTCCAG | ATTGAAGGGTGTGAGGGGAG |
| mmu HIPK2 | ATGCGTCGAAGCACTGTGAG | CCCCGCTGGCATTATTCTGAA |
| mmu convergent | CTTCCAGCACAAGAACCACA | CTCGGTACGGTTGTCTGGAT |
| mmu G3BP1 | GCTTTGTCACAGAGCCTCAAGAG | GGTTCCACTACAGGCTCCTCTA |
| mmu TNF-α | TACTGAACTTCGGGGTGATTGGTCC | CAGCCTTGTCCCTTGAAGAGAACC |
| mmu IL-1b | AGGTCCACGGGAAAGACACAGG | GGGCTGCTTCCAAACCTTTGAC |
| mmu CCL7 | GCTGCTTTCAGCATCCAAGTG | CCAGGGACACCGACTACTG |
| mmu CCL2 | GGCTCAGCCAGATGCAGTTA | ACTACAGCTTCTTTGGGACA |
| mmu NOS2 | CCAGCTCAAGAGCCAAGAAC | TCAGAGCCTCGTGGCTTTGG |
| mmu IL-10 | GCTCTTACTGACTGGCATGAG | CGCAGCTCTAGGAGCATGTG |
| mmu Arg-1 | CAGAAGAATGGAAGAGTCAG | CAGATATGCAGGGAGTCACC |
| mmu ANP | CCTGTGTACAGTGCGGTGTC | CCTAGAAGCACTGCCGTCTC |
| mmu BNP | CTGAAGGTGCTGTCCCAGAT | GTTCTTTTGTGAGGCCTTGG |
| mmu α-MHC | GGTCCACATTCTTCAGGATTCTC | GCGTTCCTTCTCTGACTTTCG |
| mmu β-MHC | TCTCCTGCTGTTTCCTTACTTGCT | CAGGCCTGTAGAAGAGCTGTACTC |
| mmu Col1a2 | CCTGGTAAAGATGGTGCC | CACCAGGTTCACCTTTCGCACC |
| mmu VEGF | TGAACACCAGCACAGGTTAC | TCTTGTTAGCTGCCTGACAC |
| mmu α-SMA | ACTACTGCCGAGCGTGAGAT | AAGGTAGACAGCGAAGCCAG |
| mmu Brca1 | CGAATCTGAGTCCCCTAAAGAGC | AAGCAACTTGACCTTGGGGTA |
| mmu NF-kB | ATGGCAGACGATGATCCCTAC | TGTTGACAGTGGTATTTCTGGTG |
| mmu GAPDH | TTCACCACCATGGAGAAGGC | GGCATGGACTGTGGTCATGA |
| mmu 18s | GTAACCCGTTGAACCCCATT | CCATCCAATCGGTAGTAGCG |
| mmu Xist | CAGGATCCTTCACATCTTCTCCACTTGAGA | CAGGATCCTTGTCTAATTCTTCTCATTGG |
| hsa circHIPK2 | CTGTGTGCTCCACCTACTTG | TACCCAGTCATGTCCCAGTTG |
| hsa HIPK2 | CCCGTGTACGAAGGTATGGC | GAAAATAGAGCCGAGTTCCAACT |
| hsa TNF-α | CTCTGGCCCAGGCAGTCAGA | GGCGTTTGGGAAGGTTGGAT |
| hsa IL-1b | CTCGCCAGTGAAATGATGGCT | TGGTGGTCGGAGATTCGTAG |
| hsa CCL2 | ATCGCCTACAGACTGCACTC | GACGGTAACGGACGTAATCAC |
| hsa IL-6 | TAGCCGCCCCACACAGACAG | GGCTGGCATTTGTGGTTGGG |
| hsa Arg-1 | TGACATCAACACACCCCTTACC | GTCCACGTCTCTCAGACCAAT |
| hsa CCL22 | ATCGCCTACAGACTGCACTC | GACGGTAACGGACGTAATCAC |
| hsa ANP | ACTCCTCTGATCGATCTGCC | CCTCCCTGGCTGTTATCTTCA |
| hsa BNP | CAAGATGGTGCAAGGGTCTG | TTCCTCTTAATGCCGCCTCA |
| hsa Col1a2 | TGACGAGACCAAGAACTG | CCATCCAAACCACTGAAACC |
| hsa α-SMA | CCTGACTGAGCGTGGCTATT | GATGAAGGATGGCTGGAACA |
| hsa IL-10 | TCAAGGCGCATGTGAACTCC | GATGTCAAACTCACTCATGGCT |
| 100-200 bp deletion | CCCCAACCCAAGCCTAC | TCCCAGTTGGAACTTGG |
| 200-400 bp deletion | ACACAAGCAGCGTGC | GATTGGCAAGGAGGTG |
| 800-900 bp deletion | CAGGTAGCCACAGCC | CGTGTGGTTCTTGTGC |

**Supplementary Table 2: Overview of clinical characteristics of patients with ischemic-oriented myocardial tissue used for validation of circHIPK2 expression.**

| **Age (years)** | **Gender** | **Underlying diseases** | **OP** |
| --- | --- | --- | --- |
| 55 | male | ICM | HTx |
| 67 | male | ICM | HTx |
| 58 | male | ICM | LVAD |
| 49 | male | ICM | HTx |
| 53 | male | ICM | HTx |

**Supplementary Table 3: Sequence information for circHIPK2 gene modulation (siRNA, shRNA and overexpression primer sequence)**

| **siRNA sequence (5'→3’)** | |
| --- | --- |
| mmu scr siR | AGGUAGUGUAAUCGCCUUG |
| hsa scr siR | UUCUCCGAACGUGUCACGUTT |
| mmu circHIPK2 siR | CAGATACTACCGGTATGGCCTCACA |
| hsa circHIPK2 siR | GUCCAGAUAUUACAGGUAUTT |
| mmu G3BP1 siR | CCAAGAUGAGGUCUUCGGUGGCUUU |
| mmu Brca1 siR | CCAAGATGCTCTGGCAGCATGTTCT |
| mmu NF-kB siR | GGGAGGAGATTTACCTTCTCTGTGA |
| **shRNA sequence (5'→3’)** | |
| Scramble | ACCTAAGGTTAAGTCGCCCTCG |
| circHIPK2 | AGATACTACCGGTATGGCCTCA |
| **pcDNA cloning primer sequence (5'→3’)** | |
| Fwd with EcoRI | CCGGAATTCGATCCTCTCCATCAAGAAGG |
| Rev with XhoI | CCGCTCGAGAAGGAGACTTGAATGTGTTT |

**Supplementary Table 4: Cytokine profiling of secretome collected from macrophages**

| **Cytokine name** | **scr siR** | **circHIPK2 siR** | **P Value** |
| --- | --- | --- | --- |
| G-CSF | 37.59 ± 3.36 | 10.57 ± 2.5 | 0.0004 |
| GM-CSF | 167.71 ± 14.52 | 5.19 ± 1.47 | 0.0006 |
| GRO/KC | 3045.15 ± 525.21 | 1889.05 ± 279.99 | 0.0282 |
| IFN-γ | 242.89 ± 64.46 | 176.42 ± 41.16 | 0.2067 |
| IL-1a | 1196.63 ± 111.49 | 78.29 ± 14.60 | 0.0001 |
| IL-1b | 136.71 ± 26.39 | 19.44 ± 4.51 | 0.0006 |
| IL-2 | 1092.49 ± 204.29 | 795.26 ± 127.74 | 0.0995 |
| IL-4 | 98.91 ± 21.09 | 75.46 ± 4.47 | 0.1328 |
| IL-5 | 109.54 ± 27.44 | 92.38 ± 20.23 | 0.4324 |
| IL-6 | 3206.41 ± 427.09 | 465 ± 58.11 | 0.0004 |
| IL-7 | 62.44 ± 7.13 | 32.53 ± 3.45 | 0.0028 |
| IL-10 | 161.98 ± 16.37 | 177.93 ± 35.25 | 0.5165 |
| IL-12(p70) | 221.89 ± 37.02 | 164.21 ± 18.88 | 0.0740 |
| IL-13 | 309.93 ± 48.32 | 259.97 ± 53.42 | 0.2959 |
| IL-17A | 22.16 ± 8.92 | 12.48 ± 2.59 | 0.2489 |
| IL-18 | 1248.34 ± 219.52 | 1171.82 ± 206.92 | 0.6831 |
| M-CSF | 705.72 ± 104.77 | 507.93 ± 64.36 | 0.0495 |
| MIP-1a | 7475.85 ± 3506.11 | 1617.74 ± 384.92 | 0.0452 |
| MIP-3a | 648.87 ± 116.09 | 856.75 ± 130.15 | 0.1079 |
| RANTES | 614.65 ± 90.24 | 443.19 ± 37.16 | 0.0383 |
| TNF-a | 5149.39 ± 729.79 | 1194.15 ± 172.95 | 0.0008 |
| VEGF | 405.69 ± 38.64 | 478.71 ± 7.26 | 0.0324 |
| MCP-1 | 2947.58 ± 278.33 | 1591.01 ± 53.74 | 0.0012 |

Cytokine concentrations were quantified using fluorescence intensity (FI) measurements. G-CSF Granulocyte colony-stimulating factor; GM-CSF Granulocyte-macrophage colony-stimulating factor; GRO/KC Chemokine ligand 1; IFN-γ Interferon gamma; IL Interleukin; M-CSF Macrophage colony-stimulating factor; MIP Macrophage inflammatory protein; RANTES Chemokine ligand 5; TNF Tumor necrosis factor; VEGF Vascular endothelial growth factor; Monocyte Chemoattractant Protein-1. Values are mean ± STDEV. p values were calculated by unpaired t.test and exact p-values are indicated.

**Supplementary Table 5: Echocardiography data from in vivo study**

| d28 post-MI | **sham** | | **MI** | |
| --- | --- | --- | --- | --- |
|  | **scr-shR** | **circHIPK2-shR (n=3)** | **scr-shR** | **circHIPK2-shR (n=7)** |
|  | **(n=3)** |  | **(n=8)** |  |
| **Body weight (g)** | 27.03 ± 1.096 | 29.43 ± 0.694 | 28.88 ± 0.917 | 29.11 ± 1.1246 |
| **LV mass (mg)** | 118.66 ± 5.316 | 126.37 ± 3.5 | 158.41 8 ± 18.226^0.001^ | 149.82 ± 8.121 |
| **Heart rate (bpm)** | 562.15 ± 39.544 | 492.27 ± 27.282 | 536.04 ± 68.554 | 527.12 ± 69.376 |
| **Infarct size (%)** | - | - | 39.68 ± 9.40 | 37.19 ± 6.09 |
| **EF (%)** | 62.04 ± 5.389 | 65.95 ± 1.340 | 24.54 ± 8.33^<0.0001^ | 30.89 ± 4.89^0.047^ |
| **EDV (μL)** | 62.60 ± 10.118 | 67.39 ± 2.31 | 115.12 ± 36.926^0.021^ | 98.88 ± 25.295 |
| **ESV (μL)** | 26.15 ± 9.172 | 24.63 ± 2.505 | 87.98 ± 37.609^0.006^ | 66.85 ± 23.245 |
| **FS (%)** | 37.82 ± 5.390 | 37.2 ± 6.683 | 10.23 ± 5.538^<0.0001^ | 17.07 ± 7.927^0.403^ |
| **LVID (mm)** | 3.54 ± 0.09 | 3.76 ± 0.856 | 4.83 ± 0.819 | 4.48 ± 0.609 |
| **LVIS (mm)** | 2.36 ± 0.412 | 2.45 ± 0.660 | 4.58 ± 0.91^0.0004^ | 3.87 ± 0.55 |
| **CO (mL/min)** | 21.69 ± 2.664 | 24.07 ± 2.50 | 13.436 ± 3.836^0.007^ | 16.74 ± 5.329 |

LV Left Ventricular; BPM Beat per minutes; EF Ejection Fraction; EDV End diastolic volume; ESV End systolic volume; FS Fraction shortening; LVID Left ventricular end diastolic dimension; LVIS Left ventricular end systolic dimension; CO cardiac output. p-values were calculated using one-way ANOVA with Tukey’s post hoc correction. Statistical significance was defined as p < 0.05; exact p-values are indicated for comparisons between sham and MI within the scramble group, and between scramble and treated groups within the MI condition.

**Supplementary Table 6. In silico prediction of splicing factors for circHIPK2 conformation**

| **Splicing factors** | **Threshold** | **Potential binding position** |
| --- | --- | --- |
| Brca1 | 1.867 | 71 |
| SC35 | 2.383 | 79 |
| SRp40 | 2.67 | 96 |
| SRp55 | 2.676 | 47 |

**Supplementary Table 7: Top 18 candidates** **selected from pulldown analysis based on higher fold change of interaction with circHIPK2**

| **Protein** | **N: Mean circHIPK22 Probe_x-y_Mean Scramble** | **p Value** |
| --- | --- | --- |
| Caprin1 | 7.40314 | 0.00014 |
| Hnrnpk | 3.58096 | 0.00079 |
| G3bp1 | 6.59992 | 0.00097 |
| Hnrnpa2b1 | 5.5688 | 0.0011 |
| Hnrnpa0 | 4.38765 | 0.0018 |
| U2af2 | 4.91026 | 0.0024 |
| Ptbp1 | 6.06211 | 0.0036 |
| Hnrnpd | 6.90787 | 0.0038 |
| Hnrnpa1 | 7.41733 | 0.0049 |
| Hnrnpab | 5.62181 | 0.0059 |
| Rbm3 | 8.23263 | 0.0074 |
| Vim | 4.15797 | 0.0091 |
| Gtf2i | 8.28136 | 0.014 |
| Fus | 9.14078 | 0.040 |
| Numa1 | 4.39486 | 0.044 |
| Prrc2c | 6.71337 | 0.047 |
| Snrnp200 | 5.03357 | 0.124 |
| Ckap5 | 3.25096 | 0.132 |

**Supplementary Table 8: An overview of the clinical information for patients from whom human heart samples were obtained for the LMS study**

| **Age (years)** | **Gender** | **Underlying diseases** | **OP** |
| --- | --- | --- | --- |
| 71 | male | Terminal heart failure | LVAD, AKE |
| 16 | female | PHT / congenital aortic valve stenosis | HTx |
| 59 | male | CAD | HTx |
| 31 | male | NCCM | HTx |
| 48 | male | DCM | HTx |

LVAD Left Ventricular Assist Device; AKE Aortic Valve Replacement; PHT Pulmonary Hypertension;

HTx Heart Transplant; CAD Coronary Artery Disease; NCCM Non-Compaction Cardiomyopathy; DCM Dilated Cardiomyopathy

**References**

1. Lu D, Chatterjee S, Xiao K, Riedel I, Huang CK, Costa A, et al. A circular RNA derived from the insulin receptor locus protects against doxorubicin-induced cardiotoxicity. *Eur Heart J* 2022;**43**:4496-4511. doi: <https://doi.org/10.1093/eurheartj/ehac337>

2. Thum T, Borlak J. Gene expression in distinct regions of the heart. *Lancet* 2000;**355**:979-983. doi: <https://doi.org/10.1016/S0140-6736(00)99016-0>

3. Viereck J, Buhrke A, Foinquinos A, Chatterjee S, Kleeberger JA, Xiao K, et al. Targeting muscle-enriched long non-coding RNA H19 reverses pathological cardiac hypertrophy. *Eur Heart J* 2020;**41**:3462-3474. doi: <https://doi.org/10.1093/eurheartj/ehaa519>

4. Garg A, Foinquinos A, Jung M, Janssen-Peters H, Biss S, Bauersachs J, et al. MiRNA-181a is a novel regulator of aldosterone-mineralocorticoid receptor-mediated cardiac remodelling. *Eur J Heart Fail* 2020;**22**:1366-1377. doi: <https://doi.org/10.1002/ejhf.1813>

5. Jung M, Ma Y, Iyer RP, DeLeon-Pennell KY, Yabluchanskiy A, Garrett MR, et al. IL-10 improves cardiac remodeling after myocardial infarction by stimulating M2 macrophage polarization and fibroblast activation. *Basic Res Cardiol* 2017;**112**:33. doi: <https://doi.org/10.1007/s00395-017-0622-5>

6. Toda G, Yamauchi T, Kadowaki T, Ueki K. Preparation and culture of bone marrow-derived macrophages from mice for functional analysis. *STAR Protoc* 2021;**2**:100246. doi: <https://doi.org/10.1016/j.xpro.2020.100246>

7. Haase A, Gohring G, Martin U. Generation of non-transgenic iPS cells from human cord blood CD34(+) cells under animal component-free conditions. *Stem Cell Res* 2017;**21**:71-73. doi: <https://doi.org/10.1016/j.scr.2017.03.022>

8. Langer LBN, Hess A, Korkmaz Z, Tillmanns J, Reffert LM, Bankstahl JP, et al. Molecular imaging of fibroblast activation protein after myocardial infarction using the novel radiotracer [(68)Ga]MHLL1. *Theranostics* 2021;**11**:7755-7766. doi: <https://doi.org/10.7150/thno.51419>

9. Heo GS, Bajpai G, Li W, Luehmann HP, Sultan DH, Dun H, et al. Targeted PET Imaging of Chemokine Receptor 2-Positive Monocytes and Macrophages in the Injured Heart. *J Nucl Med* 2021;**62**:111-114. doi: <https://doi.org/10.2967/jnumed.120.244673>

10. Hess A, Derlin T, Koenig T, Diekmann J, Wittneben A, Wang Y, et al. Molecular imaging-guided repair after acute myocardial infarction by targeting the chemokine receptor CXCR4. *Eur Heart J* 2020;**41**:3564-3575. doi: <https://doi.org/10.1093/eurheartj/ehaa598>

11. Mouton AJ, DeLeon-Pennell KY, Rivera Gonzalez OJ, Flynn ER, Freeman TC, Saucerman JJ, et al. Mapping macrophage polarization over the myocardial infarction time continuum. *Basic Res Cardiol* 2018;**113**:26. doi: <https://doi.org/10.1007/s00395-018-0686-x>

12. Piccoli MT, Gupta SK, Viereck J, Foinquinos A, Samolovac S, Kramer FL, et al. Inhibition of the Cardiac Fibroblast-Enriched lncRNA Meg3 Prevents Cardiac Fibrosis and Diastolic Dysfunction. *Circ Res* 2017;**121**:575-583. doi: <https://doi.org/10.1161/CIRCRESAHA.117.310624>

13. Haase A, Kohrn T, Fricke V, Ricci Signorini ME, Witte M, Gohring G, et al. Establishment of MHHi001-A-5, a GCaMP6f and RedStar dual reporter human iPSC line for in vitro and in vivo characterization and in situ tracing of iPSC derivatives. *Stem Cell Res* 2021;**52**:102206. doi: <https://doi.org/10.1016/j.scr.2021.102206>

14. Rodriguez Gonzalez C, Basilio-Queiros D, Neehus AL, Merkert S, Tschritter D, Unal S, et al. Human CFTR deficient iPSC-macrophages reveal impaired functional and transcriptomic response upon Pseudomonas aeruginosa infection. *Front Immunol* 2024;**15**:1397886. doi: <https://doi.org/10.3389/fimmu.2024.1397886>

15. Li Y, Xu C, Qian X, Wang G, Han C, Hua H, et al. Myeloid PTEN loss affects the therapeutic response by promoting stress granule assembly and impairing phagocytosis by macrophages in breast cancer. *Cell Death Discov* 2024;**10**:344. doi: <https://doi.org/10.1038/s41420-024-02094-0>

16. Watson SA, Scigliano M, Bardi I, Ascione R, Terracciano CM, Perbellini F. Preparation of viable adult ventricular myocardial slices from large and small mammals. *Nat Protoc* 2017;**12**:2623-2639. doi: <https://doi.org/10.1038/nprot.2017.139>

17. Watson SA, Duff J, Bardi I, Zabielska M, Atanur SS, Jabbour RJ, et al. Biomimetic electromechanical stimulation to maintain adult myocardial slices in vitro. *Nat Commun* 2019;**10**:2168. doi: <https://doi.org/10.1038/s41467-019-10175-3>

18. Fischer C, Milting H, Fein E, Reiser E, Lu K, Seidel T, et al. Long-term functional and structural preservation of precision-cut human myocardium under continuous electromechanical stimulation in vitro. *Nat Commun* 2019;**10**:117. doi: <https://doi.org/10.1038/s41467-018-08003-1>

19. Abbas N, Bentele M, Waleczek FJG, Fuchs M, Just A, Pfanne A, et al. Ex vivo modelling of cardiac injury identifies ferroptosis-related pathways as a potential therapeutic avenue for translational medicine. *J Mol Cell Cardiol* 2024;**196**:125-140. doi: <https://doi.org/10.1016/j.yjmcc.2024.09.012>

20. Abbas N, Haas JA, Xiao K, Fuchs M, Just A, Pich A, et al. Inhibition of miR-21: cardioprotective effects in human failing myocardium ex vivo. *Eur Heart J* 2024;**45**:2016-2018. doi: <https://doi.org/10.1093/eurheartj/ehae102>

21. Rosenbloom KR, Sloan CA, Malladi VS, Dreszer TR, Learned K, Kirkup VM, et al. ENCODE data in the UCSC Genome Browser: year 5 update. *Nucleic Acids Res* 2013;**41**:D56-63. doi: <https://doi.org/10.1093/nar/gks1172>

22. Chen EY, Tan CM, Kou Y, Duan Q, Wang Z, Meirelles GV, et al. Enrichr: interactive and collaborative HTML5 gene list enrichment analysis tool. *BMC Bioinformatics* 2013;**14**:128. doi: <https://doi.org/10.1186/1471-2105-14-128>

23. Chang TH, Huang HY, Hsu JB, Weng SL, Horng JT, Huang HD. An enhanced computational platform for investigating the roles of regulatory RNA and for identifying functional RNA motifs. *BMC Bioinformatics* 2013;**14 Suppl 2**:S4. doi: <https://doi.org/10.1186/1471-2105-14-S2-S4>

24. Doncheva NT, Morris JH, Gorodkin J, Jensen LJ. Cytoscape StringApp: Network Analysis and Visualization of Proteomics Data. *J Proteome Res* 2019;**18**:623-632. doi: <https://doi.org/10.1021/acs.jproteome.8b00702>

25. Neufeldt D, Schmidt A, Mohr E, Lu D, Chatterjee S, Fuchs M, et al. Circular RNA circZFPM2 regulates cardiomyocyte hypertrophy and survival. *Basic Res Cardiol* 2024;**119**:613-632. doi: <https://doi.org/10.1007/s00395-024-01048-y>

26. Cox J, Mann M. MaxQuant enables high peptide identification rates, individualized p.p.b.-range mass accuracies and proteome-wide protein quantification. *Nat Biotechnol* 2008;**26**:1367-1372. doi: <https://doi.org/10.1038/nbt.1511>

27. Cox J, Mann M. 1D and 2D annotation enrichment: a statistical method integrating quantitative proteomics with complementary high-throughput data. *BMC Bioinformatics* 2012;**13 Suppl 16**:S12. doi: <https://doi.org/10.1186/1471-2105-13-S16-S12>

28. Suarez-Arnedo A, Torres Figueroa F, Clavijo C, Arbelaez P, Cruz JC, Munoz-Camargo C. An image J plugin for the high throughput image analysis of in vitro scratch wound healing assays. *PLoS One* 2020;**15**:e0232565. doi: <https://doi.org/10.1371/journal.pone.0232565>

29. Xia J, Psychogios N, Young N, Wishart DS. MetaboAnalyst: a web server for metabolomic data analysis and interpretation. *Nucleic Acids Res* 2009;**37**:W652-660. doi: <https://doi.org/10.1093/nar/gkp356>
